# The Interactome analysis of the Respiratory Syncytial Virus protein M2-1 suggests a new role in viral mRNA metabolism post-transcription

**DOI:** 10.1038/s41598-019-51746-0

**Published:** 2019-10-24

**Authors:** Camille Bouillier, Gina Cosentino, Thibaut Léger, Vincent Rincheval, Charles-Adrien Richard, Aurore Desquesnes, Delphine Sitterlin, Sabine Blouquit-Laye, Jean-Francois Eléouët, Elyanne Gault, Marie-Anne Rameix-Welti

**Affiliations:** 1 UMR1173 INSERM, Université Paris-Saclay - UVSQ, Montigny-le-Bretonneux, France; 2 UMR7592 CNRS, Institut Jacques Monod, Université Paris Diderot, Paris, France; 3 UR892 INRA, Unité de virologie et immunologie moléculaires, Université Paris-Saclay, Jouy-en-Josas, France; 4 AP-HP, Hôpital Ambroise Paré, Laboratoire de Microbiologie, Boulogne-Billancourt, France

**Keywords:** Virus-host interactions, Proteomics

## Abstract

Human respiratory syncytial virus (RSV) is a globally prevalent negative-stranded RNA virus, which can cause life-threatening respiratory infections in young children, elderly people and immunocompromised patients. Its transcription termination factor M2-1 plays an essential role in viral transcription, but the mechanisms underpinning its function are still unclear. We investigated the cellular interactome of M2-1 using green fluorescent protein (GFP)-trap immunoprecipitation on RSV infected cells coupled with mass spectrometry analysis. We identified 137 potential cellular partners of M2-1, among which many proteins associated with mRNA metabolism, and particularly mRNA maturation, translation and stabilization. Among these, the cytoplasmic polyA-binding protein 1 (PABPC1), a candidate with a major role in both translation and mRNA stabilization, was confirmed to interact with M2-1 using protein complementation assay and specific immunoprecipitation. PABPC1 was also shown to colocalize with M2-1 from its accumulation in inclusion bodies associated granules (IBAGs) to its liberation in the cytoplasm. Altogether, these results strongly suggest that M2-1 interacts with viral mRNA and mRNA metabolism factors from transcription to translation, and imply that M2-1 may have an additional role in the fate of viral mRNA downstream of transcription.

## Introduction

Human respiratory syncytial virus (RSV) is the most common cause of respiratory infection in neonates and infants worldwide. Globally, RSV is estimated to cause 33 million cases of acute respiratory illness in children under 5 years of age, resulting in 3.2 million hospital admissions and 118 200 child deaths a year, mostly in developing countries^1^. Moreover, RSV infections in adults are increasingly associated with substantial morbidity and mortality in the elderly or at risk population, such as asthmatic and immunocompromised patients^2^.

RSV belongs to the *pneumoviridae* family of the *Mononegavirales* order^3^. Its genome consists of a single-strand negative-sense RNA tightly encapsidated by the nucleoprotein N^4^. Viral transcription and replication occur in the cytoplasm of infected cells, in virally induced cytoplasmic inclusions called inclusion bodies (IBs)^5–7^. Replication is achieved by the viral RNA dependent RNA polymerase L and its cofactor the phosphoprotein P^8^. Viral transcription requires an additional viral protein, M2-1^9^. The complex formed by L, P and M2-1 proceeds to the sequential transcription of RSV genes by a start and stop mechanism, producing capped and polyadenylated viral mRNAs^8^.

M2-1 ensures the polymerase processivity both intra- and inter-genically, preventing the synthesis of shortened mRNA and enabling transcription of downstream genes^9–11^. M2-1 is composed of four 194 amino acid chains forming a stable homo-tetrameric protein^12,13^. Each M2-1 monomer encompasses a zinc finger domain (aa 7–25) at the N terminal extremity, an α helical oligomerization domain (aa 32–49) and a large globular core domain. M2-1 interacts with the P protein and with RNA, preferentially binding to polyA rich sequences^13,14^. Both the P and RNA binding domain have been mapped: they partially overlap on the globular core domain of M2-1. It has been proposed that M2-1 associates with the polymerase complex through P interaction and then binds to the nascent viral mRNA thus dissociating from the P protein^15^. Consistent with this model, M2-1 and newly synthetized viral mRNA are concentrated together into IBs associated granules (IBAGs) shortly after transcription and are later released in the cytosol^7^. Moreover, M2-1 interaction with P enables its recruitment to RSV inclusion bodies^14^. M2-1 – P interaction also brings the phosphatase PP1 in contact with M2-1, ensuring cyclic phosphorylation and dephosphorylation of M2-1, which is needed for efficient transcription^16,17^. M2-1 was also suggested to be involved in RSV assembly by interacting with the matrix protein M^18^. This is however controversial since other reports showed that M2-1 is not required for virus-like particles (VLP) formation^19,20^. Identification of cellular proteins interacting with M2-1 could help to grasp more precisely its function, but to date no cellular partners of M2-1 have been identified.

Here, we identified potential M2-1 binding partners using affinity purified co-complexes mass spectrometry analysis on RSV infected cells. This relies on the use of a recombinant virus expressing a M2-1 protein fused to GFP^7^. Most of the candidates identified are proteins involved in mRNA metabolism, in particular mRNA maturation, stabilization and translation. We then further investigated the interaction of M2-1 and one potential M2-1 binding partner, the polyA-binding protein cytoplasmic 1 (PABPC1), a key regulator of mRNA translation and stability. In RSV infected cells, confocal microscopy analysis highlighted the co-localization of PABPC1 and M2-1 both in IBAGs and in the cytoplasm. Within IBAGs, PABPC1 exhibited the same dynamic behavior as M2-1, suggesting that both proteins remain associated with viral mRNA after its release from the IBAGs.

## Results

### Identification of potential cellular partners of M2-1

**Figure 1:**
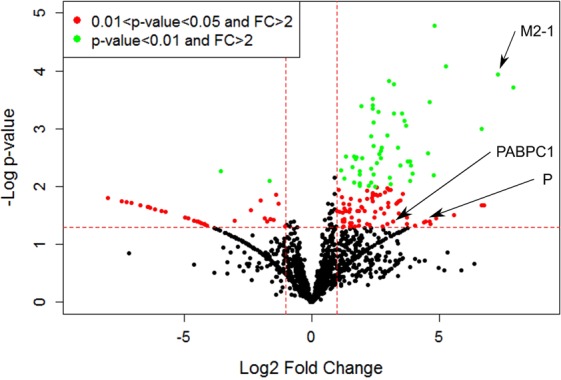
Analysis via quantitative label-free proteomics of cellular proteins associated with the viral protein M2-1-GFP during RSV infection. Co-immunoprecipitations with GFP nanobodies were realized in HEp-2 cells infected with RSV-M2-1-GFP or RSV-GFP (control). Control and experimental samples from 6 independent experiments were then analyzed by label-free quantitative proteomics processed by LC-MS/MS. P-values were computed on protein ratios with R Studio software with the R/Bioconductor software package Limma (with eBayes procedure). All proteins identified are displayed according to their fold change (FC) (x axis: log2 (FC)) as well as its statistical significance (y axis; −log10 (p-value)). Dashed red lines show chosen cutoffs: fold change = 2 [log 2 (FC) = 1] and p-value = 0.05 [log10(p-value) = 1.3]. Points highlighted in red had a fold change of more than 2 and a p-value between 0.05 and 0.01. Points highlighted in green had a fold change of more than 2 and a p-value of less than 0.01. Viral proteins M2-1 and P and cellular protein PABPC1 are highlighted with arrows.

**Figure 2:**
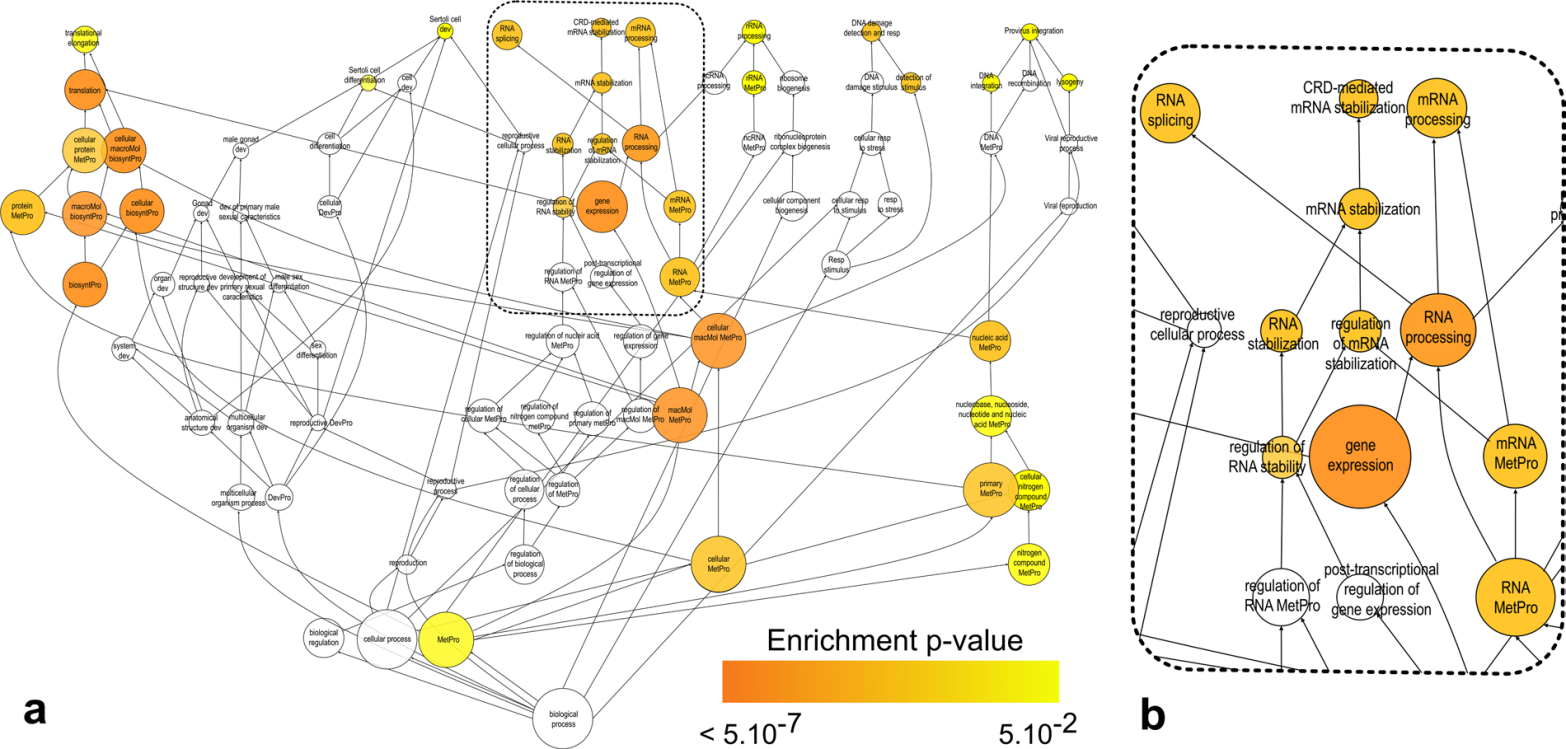
Functional groups over-represented among potential M2-1 binding partners. A gene set enrichment analysis was performed using BiNGO software on all potential M2-1 binding partners. P-values were determined by a hypergeometric test (alpha = 0.01) with a Benjamini & Hochberg correction. All GO terms related to biological processes found among this selection are displayed as circles, organized from the most general at the bottom to the most specific at the top. GO terms in an inclusion relation are linked by an arrow, with the arrow’s head pointing to the daughter GO term. Each circle’ diameter varies according to the GO term’s total size, and its color varies according to its enrichment p-value. GO terms which aren’t significantly enriched in the analysis are shown as blank circles. Abbreviations used: macromolecule = macMol, metabolic process = MetPro, development = dev, biosynthetic process = BiosyntPro. Response = resp (**a)**. Complete graph. (**b**) Area with GO terms related to RNA metabolism.

**Figure 3:**
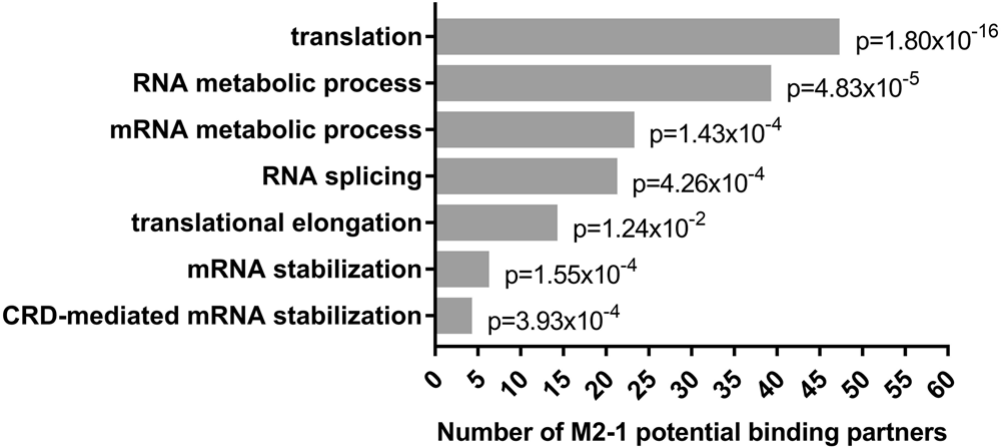
Representation of selected biological processes among potential M2-1 binding partners. A gene set enrichment analysis was performed using BiNGO software on all potential M2-1 binding partners. P-values were determined by a hypergeometric test (alpha = 0.01) with a Benjamini & Hochberg correction. For seven chosen GO terms related to biological processes, the enrichment p-values and the number of potential M2-1 binding partners featuring these GO terms are displayed in this graph.

We sought to gain insight into interactions between M2-1 and cellular proteins within infected cells. To do so, co-immunoprecipitations (IPs) of M2-1 via a GFP tag were coupled with quantitative proteomics to identify cellular proteins selectively precipitated with M2-1-GFP. We produced a recombinant RSV expressing both wild type M2-1 and M2-1 fused to GFP (RSV-M2-1-GFP^7^) and a recombinant RSV expressing free GFP (RSV-GFP). In the RSV-GFP genome, the GFP protein is inserted at the same position as the Cherry or Luc proteins of the RSV-Cherry and RSV-Luc recombinant viruses previously described^21^. HEp-2 cells were infected with these viruses in parallel at high multiplicity of infection (MOI). At 14 h post-infection (p.i) cells were lysed in the presence of RNAse A and a highly specific GFP-Trap was used to selectively precipitate the M2-1-GFP protein or the GFP protein and their interacting partners. The effectiveness of the RNAse A treatment was assessed by RT-qPCR on household gene transcripts (see Supplementary Table S1). To provide a statistically robust data set, IPs with M2-1-GFP or GFP were performed in six independent experiments. The proteins in the bound fractions were identified by Liquid Chromatography with tandem mass spectrometry (LC-MS/MS). The LC-MS/MS data were analyzed using the Mascot software search engine (Matrix Science), using only peptides with a False Discovery Rate inferior to 1% (i.e with less than 1% risk of an incorrect match between MS/MS data and peptide). Each protein was given a Mascot score based on the quality of its identification. Additionally, label free quantification was performed on proteins with at least two unique peptides. The specificity of each protein’s interaction with M2-1 was estimated by its fold change, i.e. the ratio of its abundance in the RSV-M2-1-GFP sample compared to its abundance in the RSV-GFP control. Proteins binding non-specifically to beads or to GFP would thus have a fold change of 1, and a greater fold change would indicate greater specificity. Each protein’s fold change was calculated across the 6 independent experiments thanks to a Limma t-test (with eBayes procedure) (Fig. 1). We found 137 proteins with a fold change superior to 2 with a p-value inferior to 0.05, which were selected as potential M2-1 binding partners. They are listed in the Supplementary Table S2. Among known partners of M2-1, the viral protein P was found, but not the viral protein M. Surprisingly ribosomal mitochondrial proteins were over represented (45 out of 137). This may be consistent with the detection of M2-1 in the mitochondrial fraction of infected cells^22^. A gene set enrichment analysis using the BiNGO software^23^ was realized to determine Gene Ontology (GO) terms related to biological processes over-represented in our selection (see detailed results in Supplementary Table S3). P-values of enrichment were determined by an hypergeometric test (alpha = 0.01) with a Benjamini & Hochberg correction. These functional groups are represented in Fig. 2 (and in Supplementary Fig. S1 with a higher resolution) as circles, organized from the most general functions at the bottom to the more specific at the top and linked by arrows according to the inclusion relations between them. Colored circles show the GO terms with statistically significant enrichment in our analysis, with the darkest colors indicating the smallest associated p-values. These colored circles are mainly gathered in two areas in the upper portion of the graph: one with GO terms related to translation, and the other with GO terms related to RNA metabolism and especially mRNA metabolism. All other GO terms showing significant enrichment are either very generic, and thus not very informative, or very small functional groups with a p-value barely under 0.05. Among the 137 potential M2-1 binding partners identified, 47(33.6%) were involved in translation (Fig. 3). However, 31 of them were mitochondrial ribosomal proteins, whose role in general translation should not be considered. To address this concern, we performed the enrichment analysis again, this time excluding all mitochondrial ribosomal proteins. Results are shown in the Supplementary Table S4 and Supplementary Fig. S2. The GO term “translation elongation” still appears as a significantly over-represented biological process (p = 1,36.10^−3^), but translation doesn’t appear anymore as one of the main over-represented functional groups. 39 proteins out of 137 (27.9%) potential M2-1 binding partners were proteins involved in RNA metabolism. Among these, most proteins were associated more specifically with mRNA, with the mRNA metabolic process GO term found for 23 candidates (16.4%). More precisely, the RNA splicing and mRNA stabilization subsets were over-represented, with 21 proteins (15.0%) and 6 proteins (4.3%) respectively. Interestingly, four of the five proteins of the CRD-mediated complex, which stabilizes mRNA containing the coding region instability determinant (CRD), were identified in our selection.

### The involvement of PABPC1 in RSV infection

**Figure 4:**
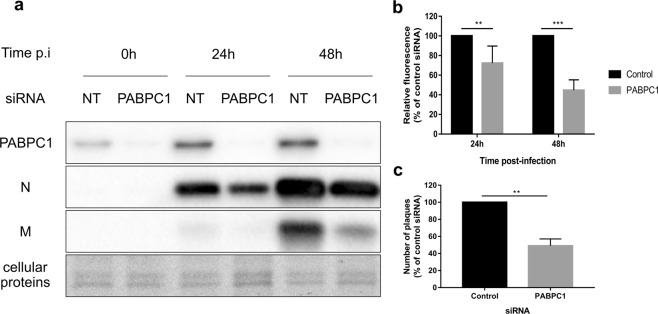
Impact of PABPC1 silencing on RSV multiplication. A549 cells were treated by siRNA PABPC1 or non-targeting siRNA (control) for 48 h and then infected with RSV-Cherry at MOI 0.05. (**a**) Cell lysates were collected at 0, 24 and 48 h p.i, subjected to SDS-PAGE and probed by antibodies directed against PABPC1, N or M. The visualization of all proteins was realized by Stain Free revelation. Full blots are available in Supplementary Fig. S4. (**b**) Cherry fluorescence was measured at 24 and 48 h post-infection and is expressed as a percentage of the values found for the control siRNA. The data was collected on 5 experiments with each point performed in triplicate. The significance was tested with a two-tailed paired t-test (alpha = 0.05) using GraphPad Prism software (**p < 0.01 (0.0073); ***p < 0.001 (0.0000025)). The normality of the data was tested with a Shapiro-Wilk normality test (alpha = 0.05) using GraphPad Prism software (p = 0.4510 & p = 0.5696). (**c**) Cell lysates were collected at 48 h p.i and subjected to a plaque titration assay. Results are expressed as the number of plaques in the 10^−5^ dilution wells, in percentage of the total found for the control siRNA. The data was collected on 3 experiments and each point was performed in duplicate. The significance was tested with a paired t-test using GraphPad Prism software (**p < 0.01 (0.0081)). The normality of the data was tested with a Shapiro-Wilk normality test (alpha = 0.05) using GraphPad Prism software (p = 0.3417).

One of the identified potential M2-1 binding partners, the cytoplasmic polyA-binding protein 1 (PABPC1) is a protein with key roles in both translation and mRNA stabilization. PABPC1 had previously been described as co-localizing with M2-1 within IBAGs^7^. We thus chose to focus on this protein in later studies. First, siRNA depletion experiments were performed to assess the role of PABPC1 in RSV multiplication. A549 cells were transfected with small interfering RNA (siRNA) targeting PABPC1 or control scrambled siRNA for 48 h and subsequently infected with a recombinant RSV carrying RSV-Cherry^21^ at a low multiplicity of inflection (M.O.I). The Cherry fluorescence was measured at 0, 24 and 48 h post-infection (p.i) to assess viral multiplication^21^ and whole cell lysates were collected. Cell viability was assessed as described in Material and Methods section. PABPC1 siRNA exhibited no cytotoxicity compared to control siRNA in these conditions and led to a decrease in PABPC1 mRNA over 80% but not to total knock-out of the protein (see Supplementary Figs S3 and S4). Consistent with this result, western blot analysis of whole cell lysates using an antibody directed against PABPC1 showed a considerable reduction in PABPC1 protein levels in cells treated with anti-PABPC1 siRNA compared with control (Fig. 4a, Supplementary Fig. S4). In contrast, cellular protein levels, observed by Stain-Free UV imaging as described in the Methods section, remained unchanged, consistent with the cell viability results. The cherry fluorescence signal in PABPC1 silenced cells was significantly reduced compared to control, with a p-value of 7.3 × 10^−3^ at 24 h p.i and 2.5 × 10^−6^ at 48 h p.i (Fig. 4b). Likewise, probing cell lysates with anti-N and M antibodies revealed a decrease of the levels of these two viral proteins in cells treated with PABPC1 siRNA compared with control at 24 and 48 h p.i (Fig. 4a, Supplementary Fig. S4). To test whether this lower infection efficiency was linked to a reduced production of virions, viruses from both control and PABPC1 siRNA treated cells were harvested at 48 h p.i and subjected to a plaque titration assay. As can be seen in Fig. 4c, the number of plaque-forming units produced dropped by 50% in cells treated by PABPC1 siRNA indicating a small but significant slowdown of viral production consistent with previous results. Together these results show that presence of PABPC1 in infected cells is required for optimal viral multiplication.

### PABPC1 interacts with M2-1 regardless of the presence of other viral proteins

**Figure 5:**
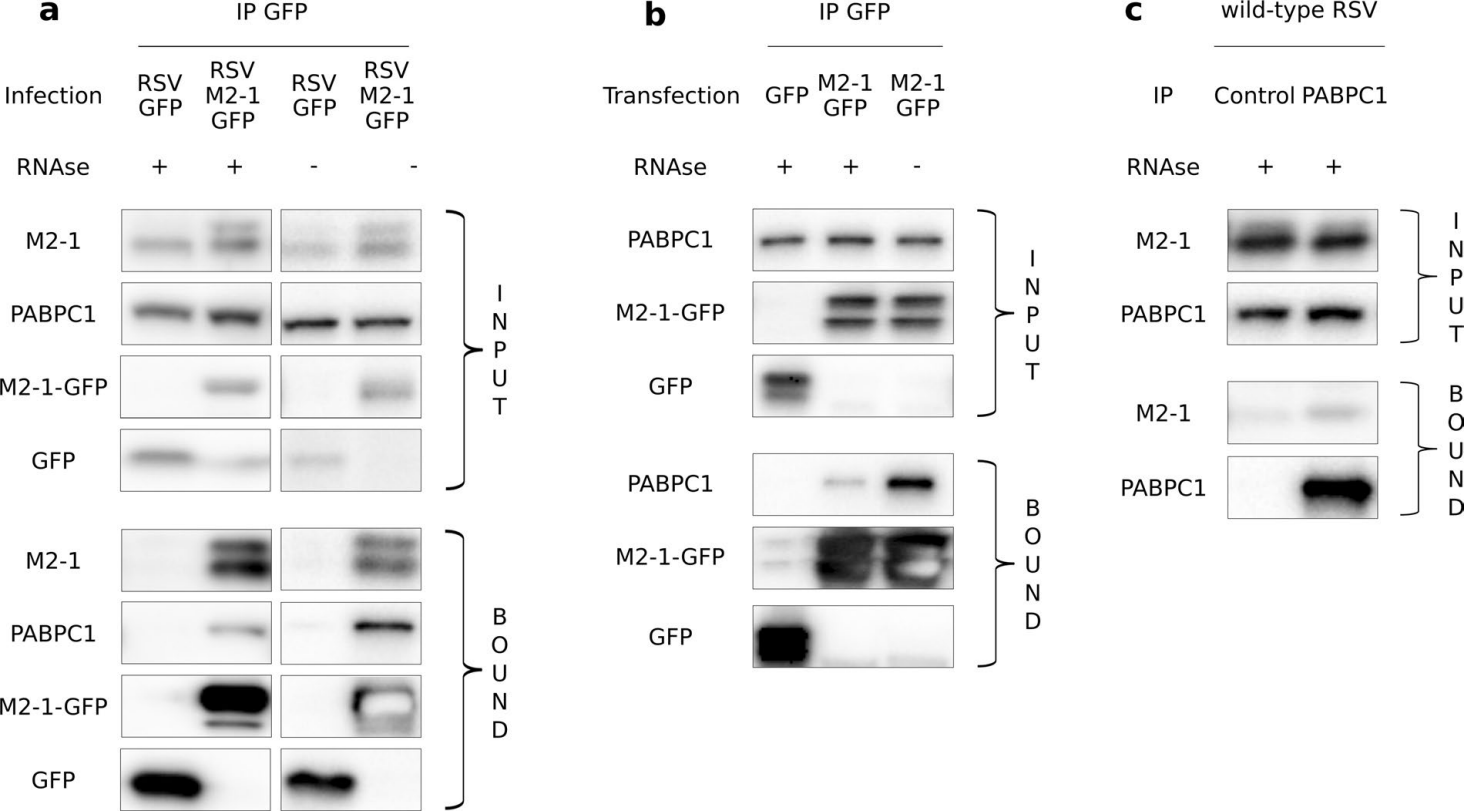
Interaction of PABPC1 with M2-1 expressed either in an infectious context or alone. (**a**) HEp-2 cells were infected with either RSV-M2-1-GFP or RSV-GFP (control) for 14 h and then subjected to co-IP with GFP antibody. RNAse (RNAse A or broad-spectrum nuclease) was added or not during the lysis step as indicated. After SDS-PAGE, immunoblot probing was then performed to detect the M2-1-GFP, GFP, PABPC1 and wild-type M2-1 proteins in the whole cell lysate (input) and in the bound fraction (bound). Full blots are available in Supplementary Fig. S5. Representative images from six to seven independent experiments are shown. (**b**) HEp-2 cells were transfected with either M2-1-GFP or GFP (control) for 24 h, and then subjected to co-IP with an anti-GFP antibody. RNAse A was added or not during the lysis step as indicated. Immunoblot probing was then performed to detect the M2-1-GFP, GFP and PABPC1 proteins. Full blots are available in Supplementary Fig. S7(a–c). Representative images from three independent experiments are shown. (**c**) HEp-2 cells were infected with wild type RSV for 14 h, and then subjected to co-IP with an antibody directed against either PABPC1 or a non-relevant protein (IMPDH2). RNAse A was added during the lysis step. M2-1 and PABPC1 were then detected by Western blotting. Full blots are available in Supplementary Fig. S7(d–e). Representative images from two independent experiments are shown.

We endeavored to validate and further characterize the PABPC1 – M2-1 interaction. As in the proteomics experiments, both RSV M2-1-GFP and RSV-GFP (control) infected HEp-2 cells were lysed and subjected to co-IP with GFP antibody. Both pre-purification cell lysates and IP samples were analyzed by western blot to reveal M2-1, PABPC1 and GFP (Fig. 5a, Supplementary Fig. S5). PABPC1 was found in the bound fraction of the M2-1-GFP sample but not of the free GFP sample (or in trace amounts) validating the interactome results (Fig. 5a). Both the phosphorylated (upper band) and the unphosphorylated (lower band) wild type M2-1 were also specifically co-immunoprecipitated with M2-1-GFP, consistent with the oligomerization of M2-1 with M2-1-GFP.

M2-1 and PABPC1 are both RNA-binding proteins^12,24^. To analyze the role of RNA in the mediation of the interaction between M2-1 and PABPC1, the co-IP experiments were performed in the presence or absence of RNAse (either RNAse A or broad spectrum nuclease). In the bound fraction, a stronger signal for PABPC1 was observed in the absence of RNAse than with RNAse treatment (Fig. 5a). Despite the non-linear nature of luminescent signals, we attempted relative quantification of these bands to obtain a very rough estimate of the amount of PABPC1 captured, in percentage of total PABPC1 in the input. 5.71% (SD: 2.94) of total PABPC1 was co-immunoprecipitated with M2-1 in absence of RNAse, against 2.08% (SD: 0.89) in presence of RNAse (see Supplementary Fig. S6). Taken together these results indicate that binding between M2-1 and PABPC1 does not require RNA as an intermediary, but that both proteins’ ability to bind to RNA either consolidates the interaction and/or leads to the capture of PABPC1 both through protein-protein and RNA-protein interactions.

Similar IP experiments were performed with BSRT7/5 cells transiently expressing M2-1-GFP and the bound proteins were revealed by immunoblotting (Fig. 5b, Supplementary Fig. S7). PABPC1 was captured in IPs with M2-1-GFP but not free GFP. Signal was stronger in the absence of RNAse A treatment as observed on infected cells. This confirms the previous results and highlights that PABPC1-M2-1 interaction does not require any other viral protein than M2-1 and can form outside of an infectious context. The mirror IP was also carried out: HEp-2 cells were infected with wild-type RSV for 14 h, then subjected to co-IP with an antibody targeting PABPC1 or a non-relevant cellular protein (control). RNAse A was added during lysis. As seen in Fig. 5c and Supplementary Fig. S7, M2-1 was found in greater quantity in the IP using PABPC1 antibody than in control. This further validates the interaction, in a setting where none of the partners were modified by tags.

### The MLLE domain of PABPC1 is responsible for its interaction with M2-1

**Figure 6:**
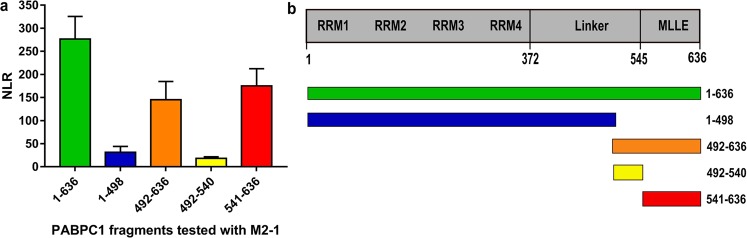
Interaction between M2-1 and various fragments of PABPC1. (**a**) The NanoLuc Two-Hybrid assay was used to detect the interaction between M2-1 and 5 fragments of PABPC1. Cells were treated with RNAse A 1 h prior to luminescence measurement. Results are displayed as a normalized luminescence ratio (NLR) of the signal of the two fusion proteins over two control protein pairs. The data was collected on 4 experiments performed in duplicates. (**b**) A schematic representation of PABPC1’s domains, with numbers representing amino acid positions of domains boundaries^45^. Below are represented the fragments of PABPC1 tested for interaction with M2-1. The numbers correspond to the positions of the first and last amino acid of each fragment.

PABPC1 is comprised of several structural domains: four RNA recognition motif (RRM) domains involved in the binding of mRNA polyA tails, a proline-rich linker and a MLLE domain involved in the binding of most cellular partners of PABPC1^24–26^. To identify which PABPC1 domain or region binds to M2-1, we performed a protein complementation assay: two complementary fragments of the NanoLuc enzyme^27^ were fused to the proteins of interest and protein-protein interactions were monitored by measuring the enzymatic activity of the reconstituted NanoLuc. The N1 and N2 complementation fragments of the NanoLuc were first fused to the entire PABPC1 and to M2-1 respectively. Fusion proteins were transiently expressed in HEK293T cells and luminescence from the reconstituted NanoLuc was measured and expressed as a normalized luminescence ratio (NLR) over control protein pairs^27^. Cell extracts were treated with RNAse A during 1 h prior to luminescence measure, to avoid the detection of mRNA mediated interactions. Strong luminescent signal was observed when the N1 and N2 fusions of the entire PABPC1 and M2-1 proteins were co-expressed, validating the interaction between PABPC1 and M2-1 (Fig. 6a).

The interaction between M2-1 and four different fragments of PABPC1 was also tested with this assay (Fig. 6b). The N-terminal fragment 1–498, spanning the 498 first amino acids of the protein, encompasses the four RRM and part of the linker. It was associated with a NLR less than 10% of the signal obtained with the whole PABPC1. In contrast, fragment 492–636, containing the rest of the linker and the MLLE domain, retained roughly 50% of the signal of the whole PABPC1. We thus focused on the C-terminal part of PABPC1 and used fragments encompassing amino acid residues 492–540 and 541–636, spanning the C-terminal part of the linker and the MLLE domain, respectively. A loss of more than 90% of the signal was observed for the fragment 492–540, while fragment 541–636 showed a NLR signal similar to the one observed with fragment 492–636. This shows that the M2-1 binding site is situated within the MLLE domain of PABPC1.

### The PABPC1/M2-1 interaction takes place both in IBAGs and in the cytoplasm of infected cells

**Figure 7:**
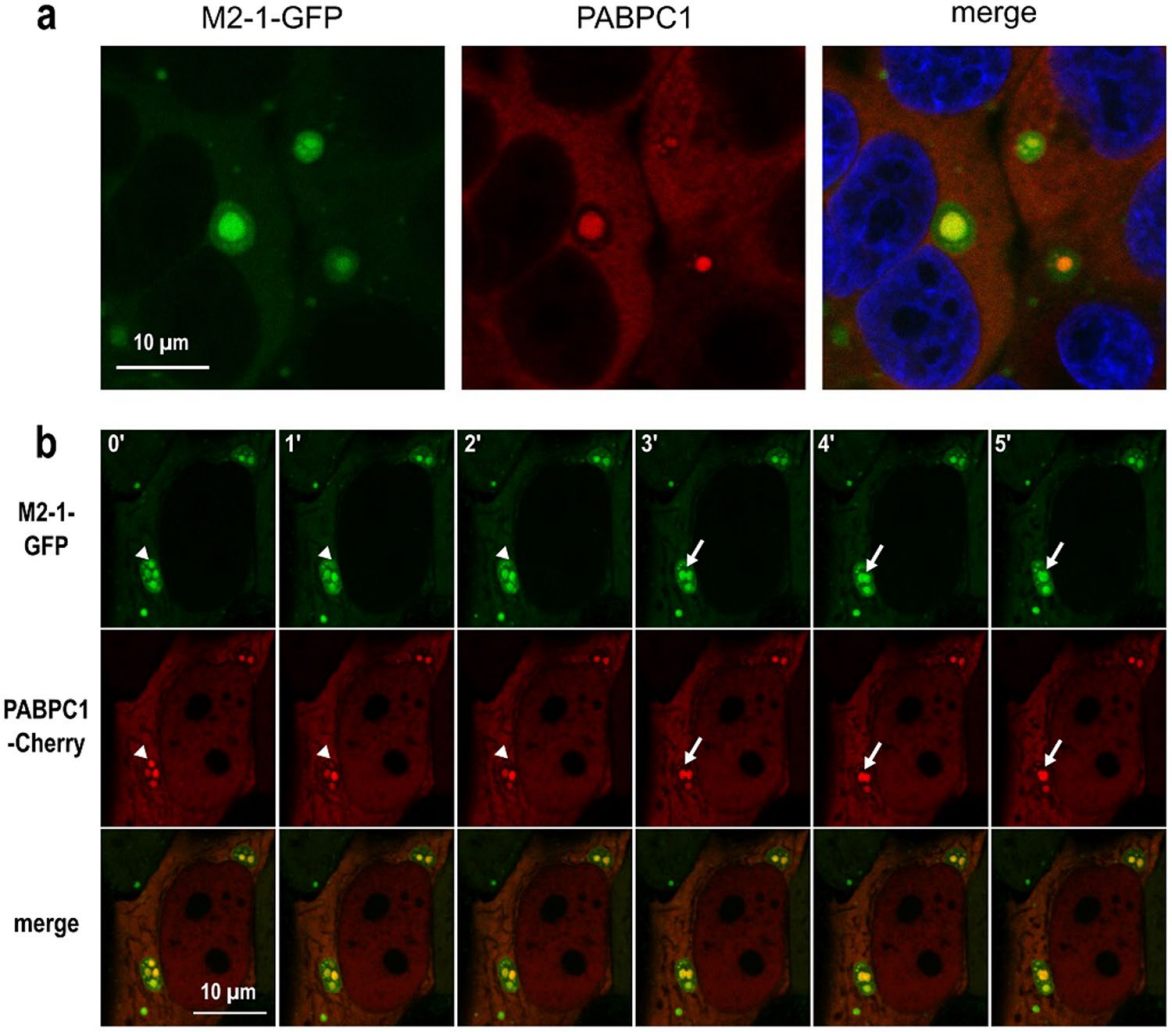
Localization of the interaction between M2-1 and PABPC1. (**a**) Cells were infected by a recombinant RSV expressing M2-1-GFP (green) and PABPC1 was localized by immunofluorescence (red). DNA was stained by Hoechst 33258 (blue). Images were taken under a Leica SP8 confocal microscope. Representative images from 3 independent experiments are shown. (**b**) Cells were simultaneously infected by RSV-M2-1-GFP and transfected with PABPC1-Cherry. At 24 h p.i., cells were imaged in a chamber heated at 37 °C, with a Olympus FV3000 confocal microscope. The M2-1-GFP protein was visualized by green fluorescence and the PABPC1-Cherry by red fluorescence. Representative images from four independent experiments are shown. White triangles and white arrows indicate M2-1 and PABPC1 signal of IBAGs undergoing disassembly and fusion, respectively.

**Figure 8:**
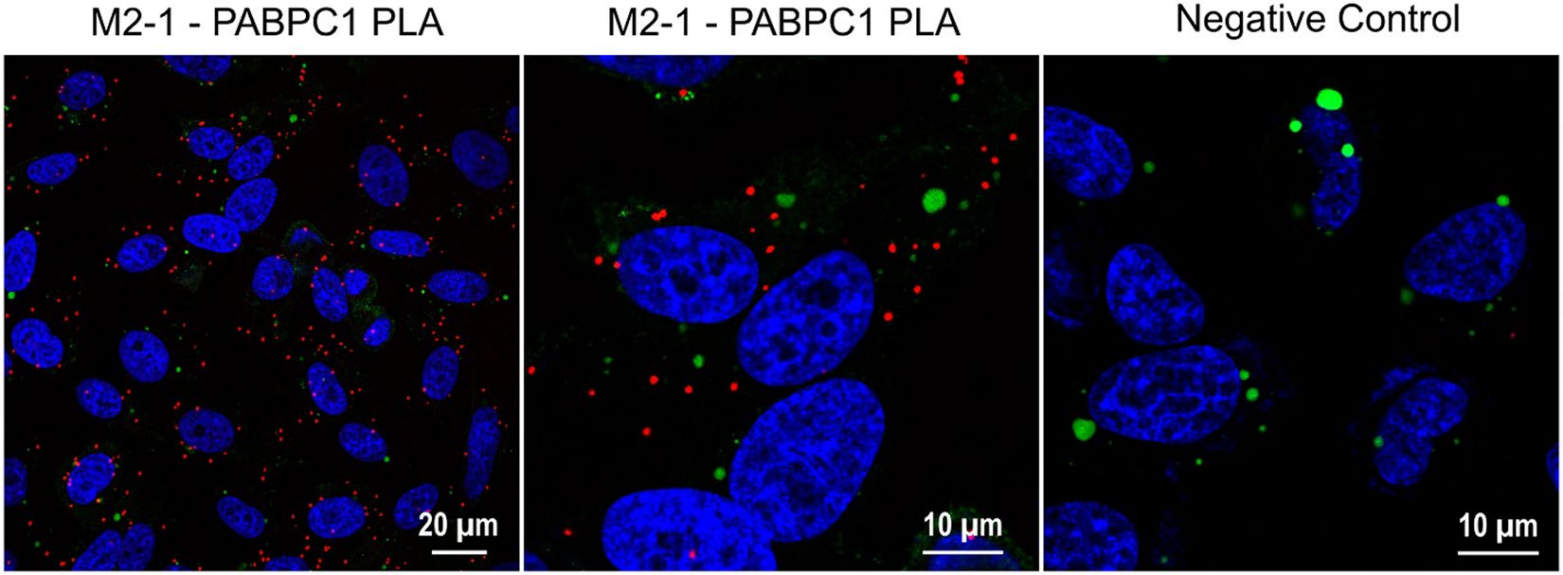
Detection of the M2-1 – PABPC1 interaction using Proximity Ligation Assay. Cells were infected with a recombinant RSV expressing L-GFP (green), fixed at 24 h p.i and subjected to a Proximity Ligation Assay (red). DNA was stained with Hoechst 33258 (blue). Primary antibodies used were directed against M2-1 and PABPC1, or M2-1 and IMPDH2 for the negative control. Images were taken under a Leica SP8 confocal microscope. Representative images from 3 independent experiments are shown.

We then investigated the subcellular localization of the M2-1 – PABPC1 interaction. M2-1 can be seen throughout the cytoplasm in infected HEp-2 cells, but accumulates in viral IBs, and more precisely in IBAGs, which are sub-compartments of IBs^7^. Staining of PABPC1 by immunofluorescence confirmed that PABPC1 can also be found throughout the cytoplasm and co-localize with M2-1 in IBAGs (Fig. 7a). PABPC1 belongs to the translation initiation complex (Wells 1998). We previously observed that eIF4G, another member of this complex, was found in IBAGs together with M2-1 and PABPC1^7^. This raises the question of whether M2-1 interacts just with PABPC1 or with the translation initiation complex as a whole. To clarify this point, M2-1-GFP was immunoprecipitated in the presence of RNAse A in RSV-M2-1-GFP infected cells, and the precipitated samples were separated by SDS-PAGE and probed by antibodies against four other members of this complex: eIF4A, eIF4E, eIF3 and eIF4G (see Supplementary Fig. S8). Consistent with the interactome results, none of those proteins are visible in the bound fraction, showing that the translation initiation complex is not co-precipitated with M2-1. To better visualize the M2-1 – PABPC1 interaction, we performed a Proximity Ligation Assay (PLA) in HEp-2 cells with antibodies directed against M2-1 and PABPC1. This test relies on close proximity of the two PLA probes triggering the hybridization of their two oligonucleotides, followed by the amplification of the reconstituted sequence and its detection by fluorescent probes. The resulting dot-shaped signal enables the visualization of the M2-1 – PABPC1 interaction within the cells. As a negative control, PLA was performed with antibodies against M2-1 and the cellular protein IMPDH2. Cells were infected by RSV-L-GFP, a recombinant virus expressing the L-GFP protein^28^ (see Methods section), to visualize IBs (in green). Red spots marking complexes of PABPC1 and M2-1 were observed in the cytoplasm, showing that the two proteins also associate outside IBs (Fig. 8). Surprisingly, no PLA signal was detected inside IBs. This could be due to an inability of the antibodies to enter the viral structures, a phenomenon often observed when immunostaining IBs induced by RSV^29^. Taken together, these observations suggest that M2-1 and PABPC1 interact both in IBAGs and in the cytosol. This could mean that their interaction is conserved as they change cellular compartments. To better understand the sequence of the association between M2-1 and PABPC1, we performed live cell imaging of transiently expressed PABPC1-Cherry and of M2-1-GFP in RSV-M2-1-GFP infected HEp-2 cells (see Supplementary Movie 1). It showed that the two proteins exhibit the same dynamic behavior when IBAGs grow and undergo fusion (Fig. 7b, white arrows). This implies that their colocalization is constant throughout their stay in IBAGs. Strikingly, the fluorescence of both M2-1 and PABPC1 in IBAGs fades concomitantly as shown on Fig. 7b (white triangles), suggesting that PABPC1 is released from IBAGs in the cytoplasm together with M2-1.

## Discussion

In this study, we investigated the interactome of the RSV protein M2-1 within infected cells, using co-immunoprecipitations coupled with a LC-MS/MS analysis. To our knowledge, this is the first study of RSV-host interactomics carried out directly in infected cells. We used a recombinant virus carrying an additional M2-1-GFP thus expressing both wild type M2-1 and M2-1-GFP fusion protein. The fusion protein M2-1-GFP showed similar efficiency to wild-type M2-1 in promoting viral transcription in minireplicon experiments and the recombinant RSV-M2-1-GFP’s growth was not significantly different from the wild type RSV’s^7^. The RSV-M2-1-GFP virus is thus a useful tool to explore M2-1 interactions with host factors during the course of viral infection. However, one drawback of this method is that the presence of a tag may prevent some proteins from binding to M2-1. In our case, though, the formation of heterotetramers M2-1:M2-1-GFP (observed in Fig. 5a) allows those proteins to be co-purified through their interaction with untagged M2-1.

A total of 137 proteins were identified as potential M2-1 binding partners. This list should not be considered exhaustive, as proteins with low abundance or weak interactions with M2-1 may not be represented. To validate these results, we chose to focus on one candidate, the polyA-binding protein cytoplasmic 1 (PABPC1). This protein plays a major role in translation as a member of the translation initiation complex, and participates in the formation of the closed loop structure taken by mRNA undergoing translation^30–33^. PABPC1 also regulates mRNA stability by protecting the polyA tail from degradation and interacting with deadenylation complexes, and is involved in miRNA and nonsense-mediated decay^34–36^. As such, it is often targeted by viruses, either to shut off cellular translation or to enhance their own proteins’ expression^37^. In particular, it is involved in several interactions with viral proteins. For example, both the rotavirus protein NSP3 and the rubella virus capsid protein bind to PABPC1 to remove it from the translation initiation complex and relocate it to the nucleus during infection, leading to an inhibition of cellular mRNA translation. On the contrary, influenza A protein NS1 binds PABPC1 without disturbing its interaction with eIF4G, and thus with the translation initiation complex. NS1 also interacts with viral mRNA, which could lead to the formation of a mRNA-NS1-PABPC1 complex which was proposed to enhance the recruitment of the translation machinery on viral mRNA^38,39^. As the RSV mRNA are both capped and polyadenylated, it is very likely that PABPC1 intervenes in their translation. Considering this, and the fact that M2-1, like influenza A NS1, binds both PABPC1 and viral mRNA, the M2-1 – PABPC1 interaction could facilitate recruitment of the translation machinery on viral mRNA. PABPC1 also plays an important role in controlling the stability of mRNAs, which could suggest that M2-1 stabilizes viral mRNAs by recruiting PABPC1. Consistent with this hypothesis, many proteins involved in regulating mRNA stability were identified as potential M2-1 interactors (Figs 2 and 3). Both hypotheses are consistent with our PABPC1 inhibition experiments, which show a significant impact of PABPC1 on RSV infection level, proteins level and virion production.

Our results clearly showed that PABPC1 was co-precipitated with M2-1 from infected cells. Quantification of Western blot signals suggest that only a small proportion of total cellular PABPC1 is involved in this interaction, which is consistent with the fact that no shut-down of cellular translation is observed in RSV infected cells. It is unclear whether the M2-1 – PABPC1 interaction is direct or not. The protein complementation assay detects close M2-1 – PABPC1 interactions that rely on the MLLE domain rather than the RNA-binding domains. This strongly suggests that the observed M2-1 – PABPC1 interaction is not due to mutual binding to the same RNA, though it probably reinforces or contributes to the interaction. This assumption is supported by the observation of an interaction between M2-1 and PABC1 in cell lysates treated by RNAse. However, since PABPC1 partially protects polyA tails against RNAse digestion, this is not an undisputable argument^34^. The M2-1 – PABPC1 interaction is also detectable in cells expressing M2-1 alone, indicating that no other RSV protein is needed as an intermediary. However, other cellular proteins could be involved. Among known PABPC1 binding partners, only hnRNP D was found as potentially interacting with M2-1 in our interactomics screen. However, its inhibition by specific siRNA had no visible impact on RSV multiplication (see Supplementary Fig. S9), suggesting that this protein does not play a role in this interaction.

The observation of the cellular colocalization of M2-1 and PABPC1 shows that these two proteins associate both within IBAGs and in the cytoplasm, and hints that they leave IBAGs together. Moreover, M2-1 is believed to bind nascent viral mRNA that concentrate in IBAGs before being released in the cytosol^7,15^. Altogether these data suggest that PABPC1 binds the M2-1-viral mRNA complex in the IBAGs and that both PABPC1 and M2-1 remain associated with viral mRNA in the cytosol after their release from IBAGs. Interestingly, the analysis of the potential M2-1 binding partners defined in this study revealed that the candidates were significantly over-represented in biological processes linked to mRNA metabolism, such as mRNA splicing, translation and stabilization. As the experiments were realized in the presence of RNAse, it is unlikely that the capture of so many RNA-binding proteins simply results from the pull-down of the entire ribonucleoprotein. This is comforted by the fact that other proteins present on mRNA, such as eIF4E, eIF4G, eIF4A and eIF3, were not precipitated with M2-1. Therefore, this result most likely indicates that M2-1, previously only known for its role in viral transcription, stays closely associated with mRNA and mRNA-binding factors (like PABPC1), throughout mRNA departure from IBAGs and release in the cytoplasm. M2-1 could thus have a hitherto unknown role in the fate of viral mRNA post-transcription, in conjunction with cellular factors involved in mRNA metabolism such as PABPC1. Like influenza A protein NS1, it could enhance the translation of RSV mRNA over cellular mRNA. It could also promote the stability of viral mRNA by consolidating the binding of cellular stabilizing factors or by disrupting the recruitment of degradation complexes. Another option is that it could lead to the recruitment of mRNA binding factors directly in IBAGs, so that when the viral mRNA is released in the cytoplasm, it is not unprotected and has all the hallmarks of a fully mature cellular mRNA. In conclusion, our results demonstrate M2-1’s involvement in the fate of viral mRNA downstream of transcription. It opens the way for the exploration of M2-1’s roles in regulation of viral mRNA maturation and stability.

## Materials and Methods

### Cells and viruses

HEp-2 cells (ATCC number CCL-23) were grown in Eagle’s minimum essential medium (MEM). BHK-21 cells (clone BSRT7/5), constitutively expressing the T7 RNA polymerase^40^, HEK293T cells (ATCC number CRL-3216), constitutively expressing the SV40 T-antigen, and A549 cells (ATCC number CCL-185) were grown in Dulbecco modified essential medium (DMEM). Media were supplemented with 10% (v/v) fetal calf serum (FCS) and antibiotics. The wild-type RSV, the RSV-GFP, the RSV-M2-1-GFP, the RSV-L-GFP and the RSV-Cherry are derived from the RSV subtype A Long strain (ATCC VR-26) and were rescued by reverse genetics as previously described^41^. Experiments were performed with viral stock amplified on HEp-2 cells at 37 °C after three to five passages^21^. Plaque assay were performed at 37 °C on HEp-2 cells using Avicel overlay as previously described^21,41^.

### Antibodies

The rabbit polyclonal anti-M2-1, anti-N and anti-M were obtained by repeated injection of purified recombinant protein produced in Escherichia coli as previously described^42^. The mouse anti-cellular proteins antibodies used in western blotting were from Santa Cruz: PABP (10E10; 0.4 µg/ml), eIF4G (A-10; 0.4 µg/ml), eIF4E (P-2; 0.4 µg/ml), eIF3η (C-5; 0.4 µg/ml) and eIF4AI/II (H-5; 0.4 µg/ml). The Santa Cruz mouse anti-PABP antibody and an abcam rabbit anti-IMPDH2 antibody (ab75790) were used in co-immunoprecipitation at a concentration of 10 µg/ml. The rabbit anti-PABP (ab21060; 2 µg/ml) and mouse anti-M2-1 (ab94805; 2 µg/ml) used in immunofluorescence staining and Duolink were from Abcam. Secondary antibodies (2 µg/ml) raised against mouse or rabbit IgG (H + L) and conjugated to Alexa Fluor 488, 594 or 647 were from Invitrogen. Secondary antibodies (0.1 µg/ml) raised against mouse or rabbit IgG (H + L) and conjugated to horseradish peroxidase were from Promega.

### Plasmids

All the viral sequences were derived from the human RSV strain Long, ATCC VR-26 (GenBank accession AY911262.1). To construct the RSV-GFP reverse genetic vector, the Cherry gene has been replaced by the GFP coding sequence in pACNR–rHRSV-cherry vector between the two MluI restriction sites^21^. The RSV-L-GFP reverse genetic vector has been derived from the pACNR–rHRSV by inserting the L-GFP coding sequence^28^ between BamHI and BstBI restriction sites. Sequences of RSV-GFP and RSV-L-GFP genomes are available on GenBank at accession numbers MK816924 and MK810782. Expression plasmids of RSV M2-1 and M2-1-GFP proteins (pM2-1 and pM2-1-GFP) have been previously described^12^. pPABPC1 was constructed by inserting the PABPC1 coding sequence in a pCI mammalian vector (Promega) between the KpnI and SalI restriction sites. pPABPC1-Cherry was constructed by inserting the Cherry coding sequence in pPABPC1 between the SalI and NotI restriction sites. The plasmids pPABPC1-C1 and pM2-1-C2 were constructed by inserting the M2-1 or PABPC1 coding sequence by Gateway recombination (Invitrogen) in pDEST-N2H-C1 and pDEST-N2H-C2 respectively^27^. In pM2-1-C2, the M2-1 coding sequence used was optimized for human expression (sequence available upon request). The pPABPC1_1-498_-N1, pPABPC1_492-636_-N1, pPABPC1_492-540_-N1 and pPABPC1_541-636_-N1 were constructed by inserting each truncated PABPC1 coding sequence in pDEST-N2H-N1, also by Gateway recombination^27^. All constructs were verified by sequencing.

### Plasmid transfection

For co-immunoprecipitations, BSR/T7-5 were transfected with Lipofectamine 2000 (Invitrogen) according to the manufacturer’s recommendations with 4 µg of plasmid. For protein complementation assays, HEK293T cells in 96-wells plates were transfected with 0.56 µL of Polyethylenimine 1 mg/mL (molecular weight 40000, Polysciences) and with 0.2 µg of plasmid.

### Protein complementation assay (N2H)

At 24 h post-transfection, cells were lysed in NanoGlo Luciferase Assay Buffer (Promega) and incubated for 1 h with 100 µg/ml of RNAse A (Thermo Scientific). After addition of an equal volume of NanoGlo Luciferase Assay Substrate (Promega) diluted 200 times in NanoGlo Luciferase Assay Buffer, luciferase activity expressed in RLU was measured using a Tecan infinite M200PRO plate reader. NLR were then calculated as previously described^27^.

### Cell infection and siRNA transfection

siRNA transfection was performed on A549 cells newly seeded in 96-wells plates^41^. Briefly, a pool of 4 different siRNA targeting the same gene (ON-TARGETplus SMARTpool, Dharmacon) was transfected simultaneously to the cell seeding, using DharmaFECT transfection reagent (Dharmacon). 0.5 µL of DharmaFECT per well were used with a final siRNA concentration of 50 nM.

### Cell viability test

Cell viability was tested using the Cell Titer Glo Luminescent Cell Viability Assay (Promega) according to the manufacturer’s recommendations. Luciferase activity expressed in RLU was measured using a Tecan infinite M200PRO plate reader.

### Co-immunoprecipitation

To perform co-immunoprecipitation using a GFP tag, HEp-2 cells infected with RSV-M2-1-GFP or transiently expressing M2-1-GFP were lysed in a co-IP lysis buffer (Tris 10 mM pH 7.5; NaCl 150 mM; IGEPAL® CA-630 0.5% (Sigma-Aldrich); antiprotease (Roche)) with or without 100 µg/ml of RNAse A (Thermo Scientific), then incubated overnight at 4 °C with GFP-Trap beads (Chromotek) according to the manufacturer’s recommendations. Beads were rinsed three times in a co-IP dilution buffer (Tris 10 mM pH 7.5; NaCl 150 mM), then either subjected to LC-MS/MS analysis or eluted in Laemmli buffer at 95 °C and analyzed by SDS-PAGE. To perform co-IP using PABP antibodies, HEp-2 cells infected with wild-type RSV were lysed in a co-IP lysis buffer (Tris 50 mM pH 7.5; NaCl 150 mM; EDTA 2 mM; DTT 1 mM; IGEPAL® CA-630 0.5% (Sigma-Aldrich); Glycerol 10%; antiprotease (Roche)) with or without RNAse. As RNAse, we used either RNAse A (Thermo Scientific) at 100 µg/ml or Pierce Universal Nuclease (Thermo Scientific) at 250U/ml. They were then incubated overnight at 4 °C with Protein A sepharose beads CL-4B (GE healthcare). The beads had previously been incubated 1 h with either mouse anti-PABP or anti-IMPDH2 antibodies and rinsed three times in PBS and one time in lysis buffer. After incubation with the lysate, beads were rinsed two times in the co-IP lysis buffer and two times in PBS, then eluted in Laemmli buffer at 95 °C and analyzed by SDS-PAGE. Western blot signals’ relative quantification was performed using the Image Lab software (Bio-Rad); for each protein, the corresponding Input signal was used as reference.

### LC-MS/MS analysis

Proteins on co-immunoprecipitation beads were incubated with NH_4_HCO_3_ 25 mM containing sequencing-grade trypsin (25 μg/mL; Promega). Peptides were desalted using ZipTip µ-C18 Pipette Tips (Millipore). Peptides mixtures were analysed by (1) a Q-Exactive Plus coupled to a Nano-LC Proxeon 1000 or by (2) an Orbitrap Fusion coupled to an UltiMate™ 3000 RSLCnano System (2) (all from Thermo Scientific). For (1), peptides were separated by chromatography with the following parameters: Acclaim PepMap100 C18 pre-column (2 cm, 75 μm i.d., 3 μm, 100 Å), Pepmap-RSLC Proxeon C18 column (50 cm, 75 μm i.d., 2 μm, 100 Å), 300 nl/min flow rate, a 98 min gradient from 95% solvent A (water, 0.1% formic acid) to 35% solvent B (100% acetonitrile, 0.1% formic acid). Peptides were analysed in the Orbitrap cell, at a resolution of 70,000, with a mass range of *m/z* 375–1500. Fragments were obtained by higher-energy collisional dissociation (HCD) activation with a collisional energy of 28%. MS/MS data were acquired in the Orbitrap cell in a Top20 mode, at a resolution of 17,500. For (2), chromatographic separation of the peptides was achieved by an Acclaim PepMap 100 C18 pre-column and a PepMap-RSLC Proxeon C18 column at a flow rate of 300 nl/min. The solvent gradient consisted of 99% solvent A (water, 0.1% (v/v) formic acid) to 45% solvent B (100% acetonitrile, 0.1% formic acid) over 55 minutes for a total gradient time of 1 hour. The Orbitrap cell analysed the peptides in full ion scan mode, with the resolution set at 120,000 with a *m/z* range of 400-1500. Collision-induced dissociation (CID) activation with a collisional energy of 35% was used for peptide fragmentation with a quadruple isolation width of 1.6 Da and a resolution of 30,000 for MS/MS. The Orbitrap cell was employed in top-speed mode in order to acquire the MS/MS data. Maximum ion accumulation times were set to 50 ms for MS acquisition and 300 ms for MS/MS acquisition in parallelization mode.

### Quantification of protein abundance variations

Peptide and protein abundance were measured using Progenesis-Qi software 4.1 (Nonlinear Dynamics Ltd, Newcastle, UK). For the identification step, all MS and MS/MS data were processed with the Proteome Discoverer software (Thermo Scientific, version 2.2) coupled to the Mascot search engine (Matrix Science, version 2.5.1). The mass tolerance was set to 7 ppm for precursor ions and 0.5 Da for fragments. The maximum number of missed cleavages was limited to two for the trypsin protease. The following variable modifications were allowed: oxidation (Met), phosphorylation (Ser, Thr, Tyr), acetylation (Protein N-term). The SwissProt database (02/2017) with the *Homo sapiens* taxonomy was used for the MS/MS identification step. Peptide Identifications were validated using a 1% FDR (False Discovery Rate) threshold calculated with the Percolator algorithm. Protein identifications were validated if at least two unique peptides were identified by protein. Protein abundance measurements were calculated according to the Hi-3 label-free quantification method. P-values were computed on protein ratios from the 6 independent experiments with R Studio software with the R/Bioconductor software package Limma (with eBayes procedure).

### Enrichment analysis of potential M2-1 binding partners

Associated Gene Ontology (GO) terms enrichment analyses and their visualization were obtained by the Bingo^23^ Cytoscape plugin (version 3.0.3) with the following parameters: hypergeometric test as statistical test with a Benjamini & Hochberg False Discovery Rate (FDR) correction and a significance level of 0.01.

### Quantitative real-time PCR

Cellular and viral mRNA were extracted from cells using QIAamp Viral RNA Mini Kit (Qiagen) according to the manufacturer’s recommendations. Genomic DNA was lysed and cDNA was synthesized from the extracts’ mRNA with random hexamer primers, using the Superscript IV VILO Mastermix with ezDNAse enzyme (Thermo Scientific) according to the manufacturer’s instructions. 4 µL of cDNA product were amplified with 0.6 µM of paired primers (presented in Supplementary Table S5) and Dynamo ColorFlash SYBR green mastermix (Thermo Scientific) according to the manufacturer’s recommendations, on a CFX96 Touch™ Real-Time PCR Detection System (Bio-Rad), following the recommended protocol. mRNA quantification was then realized using the 2^−ΔΔCT^ method with Pfaffl correction^43^.

### Immunofluorescence staining and duolink

HEp-2 cells were grown on glass coverslips, infected for 24 h then fixed with PBS-formaldehyde 4% (v/v) for 10 min at room temperature and permeabilized with PBS-BSA 1% (w/v)-Triton X-100 0.1% (v/v) for 10 min. For immunofluorescence staining, cells were incubated for 1 h with the indicated primary antibodies, and 30 min with the appropriate Alexa Fluor-conjugated secondary antibodies and Hoechst 33342 (1 μg/ml). After washing in PBS, coverslips were mounted in ProLong Diamond antifade reagent (Thermofisher). For PLA experiments, Duolink *In Situ* kit (Sigma-Aldrich) was used according to the manufacturer’s instructions. After permeabilization and blocking, cells were incubated with a mouse anti-M2-1 antibody and a rabbit anti-PABP antibody (Abcam) 60 min at room temperature in a humidity chamber. The coverslips were then incubated with corresponding secondary antibodies conjugated with PLA probes for 60 min at 37 °C. Ligation and amplification steps were performed as indicated by the manufacturer and coverslips were mounted onto slides. Z-stack image acquisitions of multi-labelled cells were performed under the WLL Leica SP8 microscope.

### Live Imaging

For time lapse microscopy, HEp-2 cells were seeded on Ibidi µ-Dish polymer coverslip bottom. Cells were transfected with pPABPC1-Cherry and infected with RSV-M2-1mGFP as described above. At 24 h p.i., cells were placed under a Olympus FV3000 confocal microscope. Z-stacks were acquired every minute during 30 min. Maximum projection was performed on Icy Software and the resulting movies were visualized on Icy Software.

## Supplementary information

Supplementary Information

Supplementary Movie S1

## Data Availability

The complete data sets are available in the PRIDE partner repository^44^ under the identification number: ProteomeXchange accession: PXD013761 Project Webpage: http://www.ebi.ac.uk/pride/archive/projects/PXD013761 FTP Download: ftp://ftp.pride.ebi.ac.uk/pride/data/archive/2019/10/PXD013761 as.raw files, Proteome Discoverer 2.2.pdResult files, associated pep.xml and xlsx files, and abundance measurements report generated by Progenesis QI.
