# Supplementary material for: The Interactome analysis of the Respiratory Syncytial Virus protein M2-1 suggests a new role in viral mRNA metabolism post-transcription
Source: Sci Rep. 2019 Oct 24;9:15258. doi: 10.1038/s41598-019-51746-0 (PMC6813310; doi:10.1038/s41598-019-51746-0)
Supplement: Supplementary file 1 — Supplementary Information [file 41598_2019_51746_MOESM1_ESM.docx]

**Supplementary Information**

**The Interactome analysis of the Respiratory Syncytial Virus protein M2-1 suggests a new role in viral mRNA metabolism post-transcription.**

Camille Bouillier^1^, Gina Cosentino^1^, Thibaut Léger^2^, Vincent Rincheval^1^, Charles-Adrien Richard^3^, Aurore Desquesnes^1^, Delphine Sitterlin^1^, Sabine Blouquit-Laye^1^, Jean-Francois Eleouet^3^, Elyanne Gault^1,4^, Marie-Anne Rameix-Welti^1,4^*

^1^UMR1173 INSERM, Université de Versailles St. Quentin, Montigny-le-Bretonneux, France

^2^UMR7592 CNRS, Institut Jacques Monod, Université Paris Diderot, Paris, France

^3^UR892 INRA, Unité de virologie et immunologie moléculaires, Université Paris-Saclay, Jouy-en-Josas, France

^4^AP-HP, Hôpital Ambroise Paré, Laboratoire de Microbiologie, Boulogne-Billancourt, France

*Corresponding Author:

Marie-Anne Rameix-Welti

marie-anne.rameix-welti@uvsq.fr

Tel: (33)170429365

Supplementary Table S1: Efficiency of RNAse A treatment

| **Buffer** | **mRNA** | **Diminution factor of mRNA level after RNAse A treatment^a^** |
| --- | --- | --- |
| Lysis buffer used in GFP-trap IP | UBC | >10^5 b^ |
|  | Actine β | 10^5^ |
|  | PABPC1 | >10^5 b^ |
| Lysis buffer used in antibody-mediated IP | UBC | >10^5 b^ |
|  | Actine β | >10^6 b^ |
|  | PABPC1 | >10^5 b^ |

^a^Cells were lysed in the conditions used for IP using GFP-trap or antibodies, in presence or absence of RNAse A, and levels of 3 mRNA, ubiquitin C, actin B and PABPC1, were measured by RT-qPCR in triplicate. Relative quantification of the target mRNA in untreated sample versus RNAse A treated sample was calculated using the following equation: $\mathbf{Diminution factor=}\frac{\mathbf{1}}{\mathbf{(Primer efficiency)}^{\mathbf{CT}\left( \mathbf{with RNAse A} \right)\mathbf{- CT (without RNAse A)}}}$. The order of magnitude of the mean is shown.

^b^No specific signal over 45 cycles was detected in the sample with RNAse A treatment. The corresponding cycle threshold (Ct) was set to 38 for the ratio calculation.

**Supplementary Table S2: Potential partners of M2-1 revealed by co-immunoprecipitation of M2-1-GFP and mass spectrometry analysis**

| **Accession** | **Protein name** | **Mascot score** | **Unique peptides** | **Fold change^a^** | **P-value^a^** |
| --- | --- | --- | --- | --- | --- |
| Q99729 | Heterogeneous nuclear ribonucleoprotein A/B | 457,2 | 12 | 38,3 | 8,37E-05 |
| - | hRSV M2-1 | 2225,8 | 30 | 158,9 | 1,14E-04 |
| P82673 | 28S ribosomal protein S35, mitochondrial | 193,5 | 12 | 3,9 | 4,11E-04 |
| Q1KMD3 | Heterogeneous nuclear ribonucleoprotein U-like protein 2 | 1931,6 | 38 | 11,7 | 5,51E-04 |
| O60506 | Heterogeneous nuclear ribonucleoprotein Q | 950,7 | 25 | 28,2 | 1,66E-05 |
| Q12905 | Interleukin enhancer-binding factor 2 | 962,1 | 16 | 8,2 | 1,49E-04 |
| Q9BYD3 | 39S ribosomal protein L4, mitochondrial | 101,1 | 4 | 9,4 | 1,70E-04 |
| Q86U42 | Polyadenylate-binding protein 2 | 323,8 | 12 | 239,2 | 1,94E-04 |
| O43390 | Heterogeneous nuclear ribonucleoprotein R | 1422,9 | 39 | 24,7 | 3,43E-04 |
| Q9NP92 | 28S ribosomal protein S30, mitochondrial | 151,1 | 4 | 5,3 | 3,89E-04 |
| Q9NZB2 | Constitutive coactivator of PPAR-gamma-like protein 1 | 325,6 | 14 | 5,3 | 4,42E-04 |
| Q14103 | Heterogeneous nuclear ribonucleoprotein D0 | 301,0 | 6 | 6,1 | 5,09E-04 |
| Q96KR1 | Zinc finger RNA-binding protein | 800,9 | 22 | 12,6 | 7,24E-04 |
| P22626 | Heterogeneous nuclear ribonucleoproteins A2/B1 | 617,3 | 15 | 5,4 | 7,78E-04 |
| O14979 | Heterogeneous nuclear ribonucleoprotein D-like | 322,9 | 6 | 7,8 | 1,29E-03 |
| P31942 | Heterogeneous nuclear ribonucleoprotein H3 | 273,1 | 11 | 6,7 | 2,12E-03 |
| Q9Y2Q9 | 28S ribosomal protein S28, mitochondrial | 210,4 | 6 | 6,3 | 2,71E-03 |
| Q08211 | ATP-dependent RNA helicase A | 1309,9 | 39 | 6,6 | 3,19E-03 |
| Q9Y2R5 | 28S ribosomal protein S17, mitochondrial | 127,1 | 2 | 3,9 | 3,22E-03 |
| P52815 | 39S ribosomal protein L12, mitochondrial | 125,9 | 4 | 3,3 | 3,34E-03 |
| Q9Y291 | 28S ribosomal protein S33, mitochondrial | 60,7 | 6 | 14,4 | 3,61E-03 |
| Q12906 | Interleukin enhancer-binding factor 3 | 955,3 | 29 | 13,5 | 3,70E-03 |
| P42285 | Superkiller viralicidic activity 2-like 2 | 611,5 | 21 | 5,0 | 4,96E-03 |
| Q00839 | Heterogeneous nuclear ribonucleoprotein U | 1665,9 | 37 | 3,7 | 6,16E-03 |
| Q8N983 | 39S ribosomal protein L43, mitochondrial | 125,2 | 6 | 8,6 | 9,01E-03 |
| Q92665 | 28S ribosomal protein S31, mitochondrial | 297,2 | 9 | 5,6 | 9,90E-03 |
| Q5T653 | 39S ribosomal protein L2, mitochondrial | 136,9 | 3 | 5,9 | 1,04E-02 |
| Q9Y3D3 | 28S ribosomal protein S16, mitochondrial | 157,0 | 3 | 6,4 | 1,38E-02 |
| Q9Y3D9 | 28S ribosomal protein S23, mitochondrial | 175,4 | 7 | 6,0 | 1,44E-02 |
| Q9Y2R9 | 28S ribosomal protein S7, mitochondrial | 276,7 | 9 | 10,5 | 2,91E-02 |
| P11940 | Polyadenylate-binding protein 1 | 328,8 | 7 | 7,8 | 4,38E-02 |
| Q8TAE8 | Growth arrest and DNA damage-inducible proteins-interacting protein 1 | 85,8 | 2 | 5,3 | 3,00E-04 |
| Q8IXM3 | 39S ribosomal protein L41, mitochondrial | 63,9 | 4 | 9,3 | 5,42E-04 |
| Q13084 | 39S ribosomal protein L28, mitochondrial | 104,2 | 5 | 12,9 | 8,77E-04 |
| Q9BZE1 | 39S ribosomal protein L37, mitochondrial | 191,1 | 4 | 5,0 | 1,35E-03 |
| P67809 | Nuclease-sensitive element-binding protein 1 | 191,8 | 6 | 5,4 | 2,01E-03 |
| P49406 | 39S ribosomal protein L19, mitochondrial | 75,9 | 3 | 10,3 | 2,15E-03 |
| Q13405 | 39S ribosomal protein L49, mitochondrial | 171,8 | 4 | 6,5 | 2,44E-03 |
| Q01780 | Exosome component 10 | 412,6 | 18 | 3,4 | 3,09E-03 |
| P09651 | Heterogeneous nuclear ribonucleoprotein A1 | 307,6 | 9 | 2,5 | 3,00E-03 |
| O95816 | BAG family molecular chaperone regulator 2 | 206,4 | 6 | 3,2 | 2,99E-03 |
| P82664 | 28S ribosomal protein S10, mitochondrial | 258,0 | 7 | 11,1 | 4,28E-03 |
| P51398 | 28S ribosomal protein S29, mitochondrial | 408,8 | 12 | 3,7 | 5,35E-03 |
| Q5BKZ1 | DBIRD complex subunit ZNF326 | 345,6 | 13 | 8,4 | 6,97E-03 |
| P82930 | 28S ribosomal protein S34, mitochondrial | 151,2 | 12 | 3,7 | 8,89E-03 |
| P82933 | 28S ribosomal protein S9, mitochondrial | 623,5 | 13 | 3,7 | 9,67E-03 |
| Q5RKV6 | Exosome complex component MTR3 | 239,4 | 4 | 4,7 | 9,75E-03 |
| Q6UXN9 | WD repeat-containing protein 82 | 235,8 | 5 | 7,8 | 1,07E-02 |
| Q9UKM9 | RNA-binding protein Raly | 307,4 | 10 | 4,5 | 1,16E-02 |
| Q9BYD1 | 39S ribosomal protein L13, mitochondrial | 112,2 | 6 | 7,3 | 1,27E-02 |
| Q9UPT8 | Zinc finger CCCH domain-containing protein 4 | 143,0 | 7 | 4,5 | 1,60E-02 |
| Q92552 | 28S ribosomal protein S27, mitochondrial | 410,2 | 14 | 2,9 | 1,64E-02 |
| Q96DV4 | 39S ribosomal protein L38, mitochondrial | 148,8 | 6 | 10,6 | 1,77E-02 |
| Q9NQT4 | Exosome complex component RRP46 | 140,9 | 2 | 7,9 | 1,89E-02 |
| **Accession** | **Protein name** | **Mascot score** | **Unique peptides** | **Fold change^a^** | **P-value^a^** |
| Q9P015 | 39S ribosomal protein L15, mitochondrial | 173,6 | 6 | 9,9 | 1,95E-02 |
| Q96EY7 | Pentatricopeptide repeat domain-containing protein 3, mitochondrial | 590,8 | 15 | 3,9 | 2,24E-02 |
| Q13868 | Exosome complex component RRP4 | 301,1 | 8 | 2,4 | 2,38E-02 |
| Q9NYK5 | 39S ribosomal protein L39, mitochondrial | 159,8 | 4 | 2,1 | 2,71E-02 |
| Q9H0U6 | 39S ribosomal protein L18, mitochondrial | 104,9 | 3 | 6,5 | 2,41E-02 |
| P07910 | Heterogeneous nuclear ribonucleoproteins C1/C2 | 859,7 | 23 | 4,2 | 2,61E-02 |
| Q08945 | FACT complex subunit SSRP1 | 143,9 | 4 | 5,7 | 2,73E-02 |
| P82912 | 28S ribosomal protein S11, mitochondrial | 148,6 | 4 | 3,6 | 2,63E-02 |
| O60783 | 28S ribosomal protein S14, mitochondrial | 96,1 | 4 | 13,3 | 3,47E-02 |
| P32969 | 60S ribosomal protein L9 | 267,9 | 5 | 4,8 | 4,54E-02 |
| P51114 | Fragile X mental retardation syndrome-related protein 1 | 325,6 | 15 | 2,8 | 4,86E-02 |
| Q9BYN8 | 28S ribosomal protein S26, mitochondrial | 120,2 | 8 | 5,8 | 3,87E-03 |
| P51991 | Heterogeneous nuclear ribonucleoprotein A3 | 384,7 | 10 | 3,0 | 4,66E-03 |
| P62701 | 40S ribosomal protein S4, X isoform | 631,5 | 12 | 2,2 | 5,16E-03 |
| Q9ULX6 | A-kinase anchor protein 8-like | 69,5 | 3 | 5,4 | 6,17E-03 |
| Q01105 | Protein SET | 260,3 | 6 | 2,4 | 7,19E-03 |
| P38159 | RNA-binding motif protein, X chromosome | 235,1 | 9 | 3,2 | 8,38E-03 |
| Q9H9J2 | 39S ribosomal protein L44, mitochondrial | 158,0 | 6 | 5,0 | 8,58E-03 |
| Q6NZY4 | Zinc finger CCHC domain-containing protein 8 | 227,3 | 7 | 8,5 | 1,14E-02 |
| - | hRSV G | 127,1 | 3 | 8,1 | 1,13E-02 |
| Q8N5N7 | 39S ribosomal protein L50, mitochondrial | 79,4 | 3 | 11,8 | 1,36E-02 |
| P82921 | 28S ribosomal protein S21, mitochondrial | 197,3 | 5 | 5,2 | 1,32E-02 |
| Q16540 | 39S ribosomal protein L23, mitochondrial | 70,2 | 1 | 5,3 | 1,42E-02 |
| Q9BVP2 | Guanine nucleotide-binding protein-like 3 | 624,5 | 18 | 2,3 | 1,51E-02 |
| Q96A35 | 39S ribosomal protein L24, mitochondrial | 79,5 | 2 | 5,1 | 1,60E-02 |
| P26447 | Protein S100-A4 | 129,3 | 6 | 2,4 | 1,98E-02 |
| Q96B26 | Exosome complex component RRP43 | 136,2 | 5 | 3,7 | 1,73E-02 |
| O43823 | A-kinase anchor protein 8 | 333,1 | 9 | 8,0 | 2,22E-02 |
| Q9Y224 | UPF0568 protein C14orf166 | 143,6 | 6 | 4,0 | 2,33E-02 |
| P62244 | 40S ribosomal protein S15a | 412,1 | 8 | 2,0 | 2,61E-02 |
| Q96GA3 | Protein LTV1 homolog | 158,6 | 7 | 2,8 | 2,75E-02 |
| P19338 | Nucleolin | 801,5 | 26 | 2,3 | 2,82E-02 |
| P40429 | 60S ribosomal protein L13a | 518,6 | 13 | 3,0 | 3,44E-02 |
| P62854 | 40S ribosomal protein S26 | 106,4 | 5 | 2,4 | 3,63E-02 |
| - | hRSV P | 220,9 | 7 | 24,7 | 4,04E-02 |
| P39023 | 60S ribosomal protein L3 | 845,2 | 22 | 2,3 | 3,99E-02 |
| P18124 | 60S ribosomal protein L7 | 653,2 | 18 | 3,0 | 4,25E-02 |
| Q02878 | 60S ribosomal protein L6 | 874,5 | 17 | 2,9 | 4,28E-02 |
| Q9BRJ2 | 39S ribosomal protein L45, mitochondrial | 71,4 | 2 | 13,3 | 4,41E-02 |
| P82675 | 28S ribosomal protein S5, mitochondrial | 228,5 | 10 | 6,9 | 4,96E-02 |
| P18827 | Syndecan-1 | 183,7 | 4 | 3,3 | 4,82E-02 |
| P50750 | Cyclin-dependent kinase 9 | 35,3 | 1 | 100,0 | 1,01E-03 |
| Q96SI9 | Spermatid perinuclear RNA-binding protein | 377,9 | 3 | 23,7 | 2,69E-03 |
| Q9H000 | Probable E3 ubiquitin-protein ligase makorin-2 | 77,0 | 2 | 14,9 | 4,26E-03 |
| Q7Z2W9 | 39S ribosomal protein L21, mitochondrial | 106,5 | 2 | 15,5 | 5,97E-03 |
| P38571 | Lysosomal acid lipase/cholesteryl ester hydrolase | 30,3 | 1 | 27,2 | 6,29E-03 |
| - | hRSV SH | 16,4 | 1 | 14,6 | 7,87E-03 |
| Q15024 | Exosome complex component RRP42 | 366,5 | 4 | 2,1 | 1,15E-02 |
| Q9UH17 | DNA dC->dU-editing enzyme APOBEC-3B | 150,0 | 4 | 5,7 | 1,39E-02 |
| P62906 | 60S ribosomal protein L10a | 180,0 | 6 | 2,8 | 2,03E-02 |
| Q6PJT7 | Zinc finger CCCH domain-containing protein 14 | 87,1 | 4 | 10,5 | 1,83E-02 |
| Q9NWU5 | 39S ribosomal protein L22, mitochondrial | 122,4 | 4 | 11,2 | 1,76E-02 |
| Q9Y580 | RNA-binding protein 7 | 103,4 | 4 | 5,3 | 1,81E-02 |
| P04040 | Catalase | 188,4 | 5 | 3,5 | 2,61E-02 |
| P82932 | 28S ribosomal protein S6, mitochondrial | 67,7 | 3 | 2,5 | 2,65E-02 |
| O43148 | mRNA cap guanine-N7 methyltransferase | 81,6 | 3 | 2,8 | 2,49E-02 |
| Q9BYC9 | 39S ribosomal protein L20, mitochondrial | 67,9 | 2 | 7,2 | 2,92E-02 |
| Q96C45 | Serine/threonine-protein kinase ULK4 | 30,5 | 1 | 47,8 | 3,10E-02 |
| Q9Y3D5 | 28S ribosomal protein S18c, mitochondrial | 69,2 | 1 | 5,5 | 3,44E-02 |
| P09874 | Poly [ADP-ribose] polymerase 1 | 371,8 | 11 | 2,3 | 3,81E-02 |
| Q9NRX2 | 39S ribosomal protein L17, mitochondrial | 51,0 | 2 | 9,1 | 4,06E-02 |
| O60814 | Histone H2B type 1-K | 547,6 | 6 | 2,4 | 4,08E-02 |
| **Accession** | **Protein name** | **Mascot score** | **Unique peptides** | **Fold change^a^** | **P-value^a^** |
| O43159 | Ribosomal RNA-processing protein 8 | 186,7 | 12 | 2,3 | 3,96E-02 |
| Q9Y5S9 | RNA-binding protein 8A | 15,6 | 1 | 3,6 | 3,95E-02 |
| Q9HD33 | 39S ribosomal protein L47, mitochondrial | 68,9 | 2 | 4,5 | 4,49E-02 |
| Q8WUY1 | Protein THEM6 | 31,0 | 1 | 25,1 | 4,50E-02 |
| P57088 | Transmembrane protein 33 | 146,3 | 3 | 3,6 | 4,66E-02 |
| P49207 | 60S ribosomal protein L34 | 133,8 | 5 | 2,3 | 4,97E-02 |
| Q5JTH9 | RRP12-like protein | 597,8 | 15 | 2,9 | 4,90E-02 |
| Q08170 | Serine/arginine-rich splicing factor 4 | 54,8 | 2 | 100,0 | 2,13E-02 |
| - | hRSV NS2 | 32,0 | 1 | 100,0 | 2,13E-02 |
| Q2YD98 | UV-stimulated scaffold protein A | 10,0 | 1 | 100,0 | 2,13E-02 |
| Q14534 | Squalene monooxygenase | 24,9 | 1 | 100,0 | 2,13E-02 |
| Q9UHR4 | Brain-specific angiogenesis inhibitor 1-associated protein 2-like protein 1 | 26,0 | 1 | 100,0 | 2,13E-02 |
| P23381 | Tryptophan--tRNA ligase, cytoplasmic | 10,0 | 1 | 100,0 | 2,13E-02 |
| Q9Y2H6 | Fibronectin type-III domain-containing protein 3A | 10,0 | 1 | 107,9 | 2,07E-02 |
| Q9UHD9 | Ubiquilin-2 | 10,0 | 1 | 100,0 | 2,13E-02 |
| P17096 | High mobility group protein HMG-I/HMG-Y | 18,6 | 1 | 29,2 | 3,52E-02 |
| P30536 | Translocator protein | 24,1 | 1 | 22,0 | 4,06E-02 |
| O75152 | Zinc finger CCCH domain-containing protein 11A | 54,2 | 3 | 21,3 | 4,12E-02 |
| Q9BQG0 | Myb-binding protein 1A | 345,4 | 15 | 2,4 | 4,87E-02 |
| P17612 | cAMP-dependent protein kinase catalytic subunit alpha | 25,9 | 1 | 16,7 | 4,71E-02 |

**^a^The fold change and p-value were estimated over 6 experiments using R Studio software with the R/Bioconductor software package Limma (with eBayes procedure). The p-value displayed is the p-value associated with the null hypothesis “fold change = 1”.**

**Supplementary Table S3: BiNGO enrichment analysis on the potential M2-1 binding partners**

| **GO-ID** | **Description** | **P-value^a^** | **Corrected p-value^b^** | **Cluster number** | **Cluster frequency** | **Total number** | **Total frequency** |
| --- | --- | --- | --- | --- | --- | --- | --- |
| 10467 | gene expression | 3E-23 | 7,7E-21 | 80 | 57,1429 | 417 | 20,4713 |
| 6412 | translation | 1,4E-18 | 1,8E-16 | 47 | 33,5714 | 175 | 8,59107 |
| 34645 | cellular macromolecule biosynthetic process | 2,1E-14 | 1,6E-12 | 54 | 38,5714 | 281 | 13,7948 |
| 9059 | macromolecule biosynthetic process | 4,7E-14 | 3,3E-12 | 54 | 38,5714 | 286 | 14,0403 |
| 44260 | cellular macromolecule metabolic process | 3,6E-13 | 2,2E-11 | 93 | 66,4286 | 758 | 37,2116 |
| 43170 | macromolecule metabolic process | 1,7E-11 | 8,5E-10 | 93 | 66,4286 | 801 | 39,3225 |
| 44249 | cellular biosynthetic process | 6E-09 | 2,7E-07 | 57 | 40,7143 | 411 | 20,1767 |
| 9058 | biosynthetic process | 4,7E-08 | 1,7E-06 | 57 | 40,7143 | 433 | 21,2568 |
| 6396 | RNA processing | 2,5E-07 | 8,5E-06 | 35 | 25 | 213 | 10,4566 |
| 44237 | cellular metabolic process | 3,9E-07 | 1,3E-05 | 98 | 70 | 1013 | 49,73 |
| 44238 | primary metabolic process | 6,5E-07 | 2E-05 | 96 | 68,5714 | 991 | 48,65 |
| 42769 | DNA damage response, detection of DNA damage | 1,4E-06 | 4,4E-05 | 5 | 3,57143 | 5 | 0,24546 |
| 16070 | RNA metabolic process | 1,6E-06 | 4,8E-05 | 39 | 27,8571 | 271 | 13,3039 |
| 43489 | RNA stabilization | 2,4E-06 | 6,7E-05 | 6 | 4,28571 | 8 | 0,39273 |
| 48255 | mRNA stabilization | 2,4E-06 | 6,7E-05 | 6 | 4,28571 | 8 | 0,39273 |
| 90304 | nucleic acid metabolic process | 2,6E-06 | 7,1E-05 | 46 | 32,8571 | 353 | 17,3294 |
| 16071 | mRNA metabolic process | 5,9E-06 | 0,00014 | 23 | 16,4286 | 125 | 6,13648 |
| 43487 | regulation of RNA stability | 6,7E-06 | 0,00016 | 6 | 4,28571 | 9 | 0,44183 |
| 43488 | regulation of mRNA stability | 6,7E-06 | 0,00016 | 6 | 4,28571 | 9 | 0,44183 |
| 44267 | cellular protein metabolic process | 1,4E-05 | 0,00029 | 54 | 38,5714 | 470 | 23,0731 |
| 70934 | CRD-mediated mRNA stabilization | 2,1E-05 | 0,00039 | 4 | 2,85714 | 4 | 0,19637 |
| 8380 | RNA splicing | 2,4E-05 | 0,00043 | 21 | 15 | 117 | 5,74374 |
| 51606 | detection of stimulus | 2,7E-05 | 0,00047 | 5 | 3,57143 | 7 | 0,34364 |
| 6397 | mRNA processing | 4,1E-05 | 0,0007 | 20 | 14,2857 | 112 | 5,49828 |
| 19538 | protein metabolic process | 7,2E-05 | 0,00119 | 55 | 39,2857 | 508 | 24,9386 |
| 8152 | metabolic process | 0,00012 | 0,00189 | 98 | 70 | 1119 | 54,9337 |
| 6139 | nucleotide and nucleic acid metabolic process | 0,00074 | 0,01026 | 46 | 32,8571 | 436 | 21,404 |
| 6414 | translational elongation | 0,00091 | 0,01242 | 14 | 10 | 81 | 3,97644 |
| 19047 | provirus integration | 0,00287 | 0,03403 | 3 | 2,14286 | 5 | 0,24546 |
| 15074 | DNA integration | 0,00287 | 0,03403 | 3 | 2,14286 | 5 | 0,24546 |
| 30069 | lysogeny | 0,00287 | 0,03403 | 3 | 2,14286 | 5 | 0,24546 |
| 34641 | cellular nitrogen compound metabolic process | 0,00288 | 0,03403 | 47 | 33,5714 | 476 | 23,3677 |
| 6364 | rRNA processing | 0,00342 | 0,03995 | 10 | 7,14286 | 55 | 2,70005 |
| 16072 | rRNA metabolic process | 0,00393 | 0,04532 | 10 | 7,14286 | 56 | 2,74914 |
| 6807 | nitrogen compound metabolic process | 0,00431 | 0,04865 | 47 | 33,5714 | 485 | 23,8095 |
| 60008 | Sertoli cell differentiation | 0,00469 | 0,04875 | 2 | 1,42857 | 2 | 0,09818 |
| 60009 | Sertoli cell development | 0,00469 | 0,04875 | 2 | 1,42857 | 2 | 0,09818 |

^a^P-value determined by a hypergeometric test (alpha=0.01) without correcting for multiple tests

^b^P-value determined by a hypergeometric test (alpha=0.01) with a Benjamini & Hochberg correction.


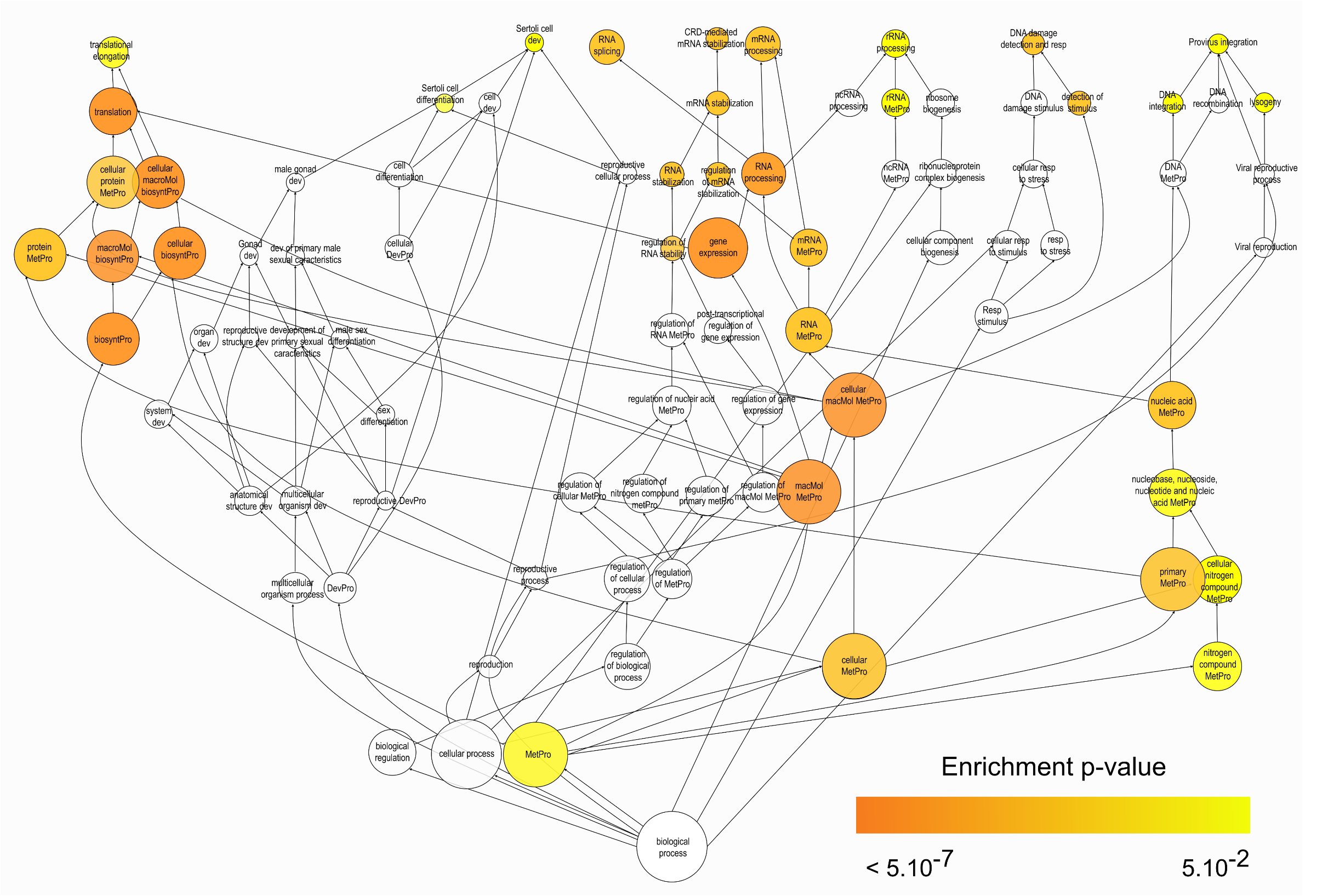


Supplementary Figure S1: Functional groups over-represented among potential M2-1 binding partners.

A gene set enrichment analysis was performed using BiNGO software on all potential M2-1 binding partners. P-values were determined by a hypergeometric test (alpha=0.01) with a Benjamini & Hochberg correction. All GO terms related to biological processes found among this selection are displayed as circles, organized from the most general at the bottom to the most specific at the top. GO terms in an inclusion relation are linked by an arrow, with the arrow’s head pointing to the daughter GO term. Each circle’ diameter varies according to the GO term’s total size, and its color varies according to its enrichment p-value. GO terms which aren’t significantly enriched in the analysis are shown as blank circles. Abbreviations used: macromolecule=macMol, metabolic process=MetPro, development=dev, biosynthetic process=BiosyntPro. Response=resp.

**Supplementary Table S4: BiNGO enrichment analysis on the potential M2-1 binding partners, excluding ribosomal mitochondrial proteins**

| **GO-ID** | **Description** | **P-value^a^** | **Corrected p-value^b^** | **Cluster number** | **Cluster frequency** | **Total number** | **Total frequency** |
| --- | --- | --- | --- | --- | --- | --- | --- |
| 3676 | nucleic acid binding | 3.08E-21 | 2.77E-18 | 58 | 72.5% | 664 | 23.2% |
| 3723 | RNA binding | 4.25E-20 | 1.91E-17 | 44 | 55.0% | 365 | 12.7% |
| 30529 | ribonucleoprotein complex | 3.26E-12 | 9.75E-10 | 33 | 41.2% | 325 | 11.3% |
| 44428 | nuclear part | 6.10E-12 | 1.37E-09 | 45 | 56.2% | 615 | 21.4% |
| 6396 | RNA processing | 1.75E-10 | 2.70E-08 | 29 | 36.2% | 290 | 10.1% |
| 10467 | gene expression | 1.80E-10 | 2.70E-08 | 40 | 50.0% | 541 | 18.9% |
| 31981 | nuclear lumen | 8.52E-10 | 1.09E-07 | 36 | 45.0% | 467 | 16.3% |
| 16070 | RNA metabolic process | 9.89E-10 | 1.11E-07 | 32 | 40.0% | 376 | 13.1% |
| 30530 | heterogeneous nuclear ribonucleoprotein complex | 2.82E-09 | 2.82E-07 | 8 | 10.0% | 16 | 0.5% |
| 90304 | nucleic acid metabolic process | 2.20E-08 | 1.94E-06 | 35 | 43.7% | 498 | 17.4% |
| 16071 | mRNA metabolic process | 2.37E-08 | 1.94E-06 | 20 | 25.0% | 174 | 6.0% |
| 5634 | nucleus | 2.85E-08 | 2.00E-06 | 55 | 68.7% | 1103 | 38.5% |
| 43489 | RNA stabilization | 3.11E-08 | 2.00E-06 | 6 | 7.5% | 9 | 0.3% |
| 48255 | mRNA stabilization | 3.11E-08 | 2.00E-06 | 6 | 7.5% | 9 | 0.3% |
| 178 | exosome (RNase complex) | 7.61E-08 | 4.02E-06 | 6 | 7.5% | 10 | 0.3% |
| 43487 | regulation of RNA stability | 7.61E-08 | 4.02E-06 | 6 | 7.5% | 10 | 0.3% |
| 43488 | regulation of mRNA stability | 7.61E-08 | 4.02E-06 | 6 | 7.5% | 10 | 0.3% |
| 8380 | RNA splicing | 1.30E-07 | 6.50E-06 | 18 | 22.5% | 156 | 5.4% |
| 5730 | nucleolus | 2.30E-07 | 1.09E-05 | 22 | 27.5% | 238 | 8.3% |
| 70934 | CRD-mediated mRNA stabilization | 5.68E-07 | 2.43E-05 | 4 | 5.0% | 4 | 0.1% |
| 70937 | CRD-mediated mRNA stability complex | 5.68E-07 | 2.43E-05 | 4 | 5.0% | 4 | 0.1% |
| 6397 | mRNA processing | 7.89E-07 | 3.22E-05 | 17 | 21.2% | 157 | 5.4% |
| 31974 | membrane-enclosed lumen | 9.70E-07 | 3.72E-05 | 37 | 46.2% | 631 | 22.0% |
| 8408 | 3'-5' exonuclease activity | 9.95E-07 | 3.72E-05 | 6 | 7.5% | 14 | 0.4% |
| 70013 | intracellular organelle lumen | 1.36E-06 | 4.87E-05 | 36 | 45.0% | 611 | 21.3% |
| 43233 | organelle lumen | 1.54E-06 | 5.31E-05 | 36 | 45.0% | 614 | 21.4% |
| 5681 | spliceosomal complex | 1.69E-06 | 5.43E-05 | 12 | 15.0% | 82 | 2.8% |
| 175 | 3'-5'-exoribonuclease activity | 1.74E-06 | 5.43E-05 | 5 | 6.2% | 9 | 0.3% |
| 3677 | DNA binding | 1.75E-06 | 5.43E-05 | 23 | 28.7% | 289 | 10.1% |
| 6139 | nucleobase, nucleoside, nucleotide and nucleic acid metabolic process | 2.41E-06 | 7.16E-05 | 35 | 43.7% | 597 | 20.8% |
| 32991 | macromolecular complex | 2.47E-06 | 7.16E-05 | 49 | 61.2% | 1026 | 35.8% |
| 44260 | cellular macromolecule metabolic process | 3.03E-06 | 8.51E-05 | 48 | 60.0% | 999 | 34.9% |
| 3729 | mRNA binding | 3.35E-06 | 8.74E-05 | 8 | 10.0% | 35 | 1.2% |
| 4532 | exoribonuclease activity | 3.41E-06 | 8.74E-05 | 5 | 6.2% | 10 | 0.3% |
| 16896 | exoribonuclease activity, producing 5'-phosphomonoesters | 3.41E-06 | 8.74E-05 | 5 | 6.2% | 10 | 0.3% |
| 34641 | cellular nitrogen compound metabolic process | 5.72E-06 | 1.43E-04 | 36 | 45.0% | 647 | 22.6% |
| 16796 | exonuclease activity, active with either ribo- or deoxyribonucleic acids and producing 5'-phosphomonoesters | 6.11E-06 | 1.48E-04 | 5 | 6.2% | 11 | 0.3% |
| 6807 | nitrogen compound metabolic process | 9.65E-06 | 2.28E-04 | 36 | 45.0% | 661 | 23.1% |
| 4527 | exonuclease activity | 1.12E-05 | 2.59E-04 | 6 | 7.5% | 20 | 0.6% |
| 43170 | macromolecule metabolic process | 1.73E-05 | 3.87E-04 | 48 | 60.0% | 1055 | 36.8% |
| 6414 | translational elongation | 6.21E-05 | 1.36E-03 | 10 | 12.5% | 81 | 2.8% |
| 70717 | poly-purine tract binding | 8.26E-05 | 1.72E-03 | 3 | 3.7% | 4 | 0.1% |
| 8143 | poly(A) RNA binding | 8.26E-05 | 1.72E-03 | 3 | 3.7% | 4 | 0.1% |
| 44446 | intracellular organelle part | 1.15E-04 | 2.35E-03 | 57 | 71.2% | 1448 | 50.6% |
| 3727 | single-stranded RNA binding | 1.61E-04 | 3.22E-03 | 4 | 5.0% | 11 | 0.3% |
| 44422 | organelle part | 1.66E-04 | 3.23E-03 | 57 | 71.2% | 1463 | 51.1% |
| 10608 | posttranscriptional regulation of gene expression | 2.06E-04 | 3.94E-03 | 9 | 11.2% | 76 | 2.6% |
| 4540 | ribonuclease activity | 3.42E-04 | 6.39E-03 | 5 | 6.2% | 23 | 0.8% |
| 42162 | telomeric DNA binding | 3.96E-04 | 7.16E-03 | 3 | 3.7% | 6 | 0.2% |
| 10468 | regulation of gene expression | 3.99E-04 | 7.16E-03 | 23 | 28.7% | 402 | 14.0% |
| 51252 | regulation of RNA metabolic process | 4.92E-04 | 8.67E-03 | 14 | 17.5% | 186 | 6.5% |
| 43228 | non-membrane-bounded organelle | 5.42E-04 | 9.19E-03 | 37 | 46.2% | 821 | 28.6% |
| 43232 | intracellular non-membrane-bounded organelle | 5.42E-04 | 9.19E-03 | 37 | 46.2% | 821 | 28.6% |
| 5488 | binding | 6.20E-04 | 1.03E-02 | 75 | 93.7% | 2299 | 80.3% |
| 6364 | rRNA processing | 6.70E-04 | 1.09E-02 | 8 | 10.0% | 71 | 2.4% |
| 16072 | rRNA metabolic process | 7.37E-04 | 1.18E-02 | 8 | 10.0% | 72 | 2.5% |
| 60008 | Sertoli cell differentiation | 7.72E-04 | 1.20E-02 | 2 | 2.5% | 2 | 0.0% |
| 60009 | Sertoli cell development | 7.72E-04 | 1.20E-02 | 2 | 2.5% | 2 | 0.0% |
| 4518 | nuclease activity | 9.29E-04 | 1.41E-02 | 6 | 7.5% | 42 | 1.4% |
| 44238 | primary metabolic process | 1.38E-03 | 2.07E-02 | 50 | 62.5% | 1300 | 45.4% |
| 166 | nucleotide binding | 1.96E-03 | 2.89E-02 | 29 | 36.2% | 624 | 21.8% |
| 22626 | cytosolic ribosome | 2.11E-03 | 3.06E-02 | 7 | 8.7% | 66 | 2.3% |
| 71204 | histone pre-mRNA 3'end processing complex | 2.28E-03 | 3.24E-02 | 2 | 2.5% | 3 | 0.1% |
| 6401 | RNA catabolic process | 2.36E-03 | 3.31E-02 | 4 | 5.0% | 21 | 0.7% |
| 44237 | cellular metabolic process | 2.45E-03 | 3.39E-02 | 50 | 62.5% | 1328 | 46.4% |
| 3697 | single-stranded DNA binding | 2.83E-03 | 3.84E-02 | 4 | 5.0% | 22 | 0.7% |
| 19219 | regulation of nucleobase, nucleoside, nucleotide and nucleic acid metabolic process | 3.01E-03 | 4.03E-02 | 20 | 25.0% | 380 | 13.2% |
| 3735 | structural constituent of ribosome | 3.05E-03 | 4.03E-02 | 10 | 12.5% | 131 | 4.5% |
| 5654 | nucleoplasm | 3.52E-03 | 4.54E-02 | 16 | 20.0% | 278 | 9.7% |
| 31323 | regulation of cellular metabolic process | 3.57E-03 | 4.54E-02 | 25 | 31.2% | 528 | 18.4% |
| 60255 | regulation of macromolecule metabolic process | 3.59E-03 | 4.54E-02 | 24 | 30.0% | 499 | 17.4% |
| 51171 | regulation of nitrogen compound metabolic process | 3.74E-03 | 4.67E-02 | 20 | 25.0% | 387 | 13.5% |

^a^P-value determined by a hypergeometric test (alpha=0.01) without correcting for multiple tests

**^b^P-value determined by a hypergeometric test (alpha=0.01) with a Benjamini & Hochberg correction**


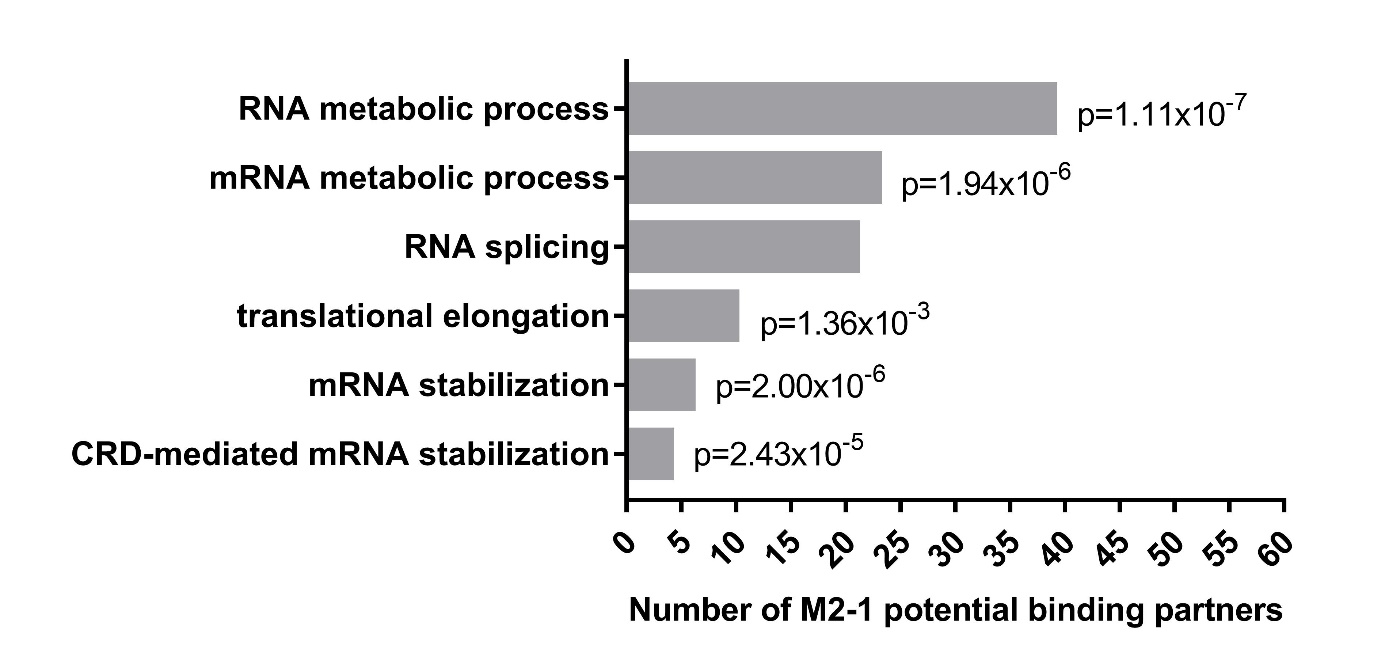


Supplementary Figure S2: Representation of selected biological processes among potential M2-1 binding partners, excluding mitochondrial ribosomal proteins.

A gene set enrichment analysis was performed using BiNGO software on all potential M2-1 binding partners, excluding mitochondrial ribosomal proteins. P-values were determined by a hypergeometric test (alpha=0.01) with a Benjamini & Hochberg correction. For six chosen GO terms related to biological processes, the enrichment p-values and the number of potential M2-1 binding partners featuring these GO terms are displayed in this graph.


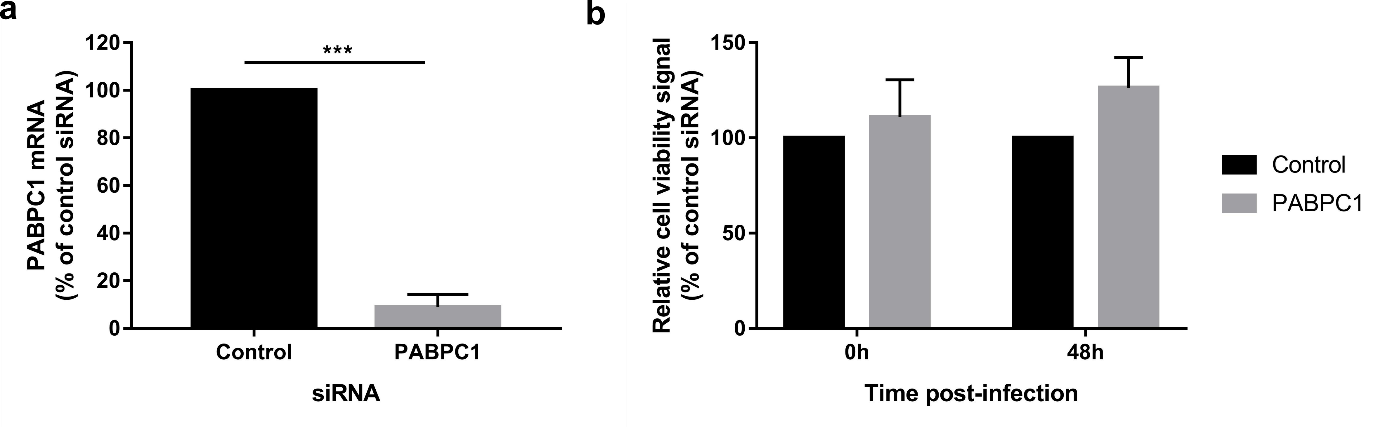


Supplementary Figure S3: PABPC1 silencing by siRNA.

A549 cells were treated by siRNA targeting PABPC1 and non-targeting siRNA (control) and infected after 48h by RSV-Cherry at low MOI. a. To verify siRNA silencing efficiency, PABPC1 mRNA levels were quantified by RT-qPCR in cells treated by siRNA directed against PABPC1 or non-targeting siRNA (control) at 0h p.i. The data shown (mean $\boldsymbol{\pm}$ s.d.) represents 6 independent experiments. The significance was tested with a two-tailed paired t-test (alpha=0.05) using GraphPad Prism software (*** p<0.001 (0.0002)). The normality of the data was tested with a Shapiro-Wilk normality test (alpha=0.05) using GraphPad Prism software (p=0.6539). b. Cell viability was measured at 0 and 48h p.i using Cell Titer Glo kit (Promega). The cell viability signal was obtained as a luminescence measure, and expressed as a percentage of the control siRNA's signal. The data shown (mean $\boldsymbol{\pm}$ s.d.) represents 4 independent experiments, with each point performed in duplicate. No significant difference between the two conditions was found by a two-tailed paired t-test (alpha=0.05) using GraphPad Prism software (p=0.3116 & p=0.0790). The normality of the data was tested with a Shapiro-Wilk normality test (alpha=0.05) using GraphPad Prism software (p=0.1895 & p=0.2528).


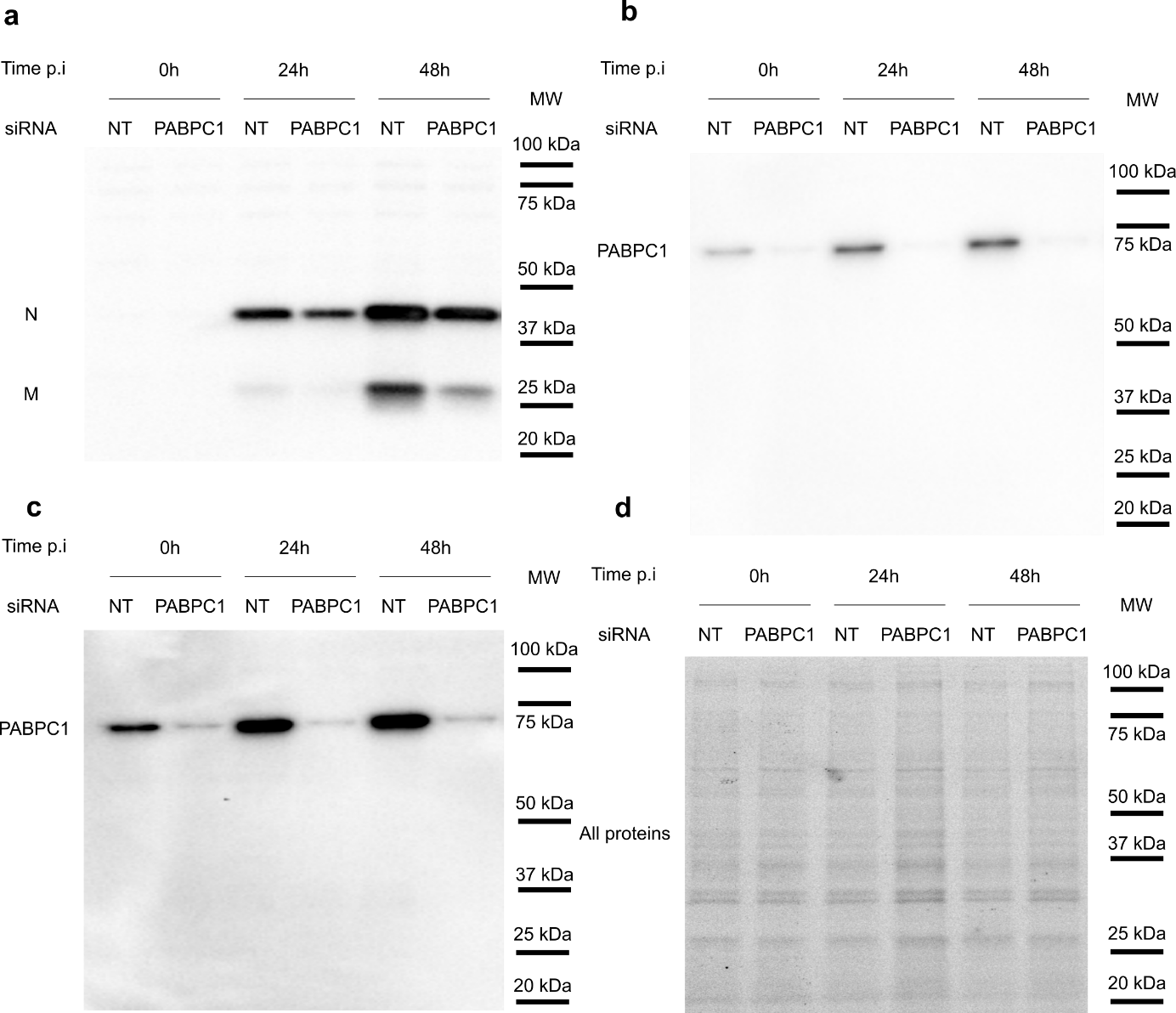


Supplementary Figure S4: Impact of PABPC1 silencing on RSV multiplication (complement to Figure 4a)

A549 cells were treated by siRNA PABPC1 or non-targeting siRNA (control) for 48h and then infected with RSV-Cherry at MOI 0.05. Cell lysates were collected at 0, 24 and 48h p.i and subjected to SDS-PAGE. a. The membrane was probed by antibodies directed against N and M. b-c. The membrane was probed by an antibody directed against PABPC1. d. The visualization of all proteins was realized by Stain Free revelation. a-b-d. Antibody signals were visualized by luminescence, at an exposition where no pixel was saturated. c. Antibody signals were visualized by luminescence, at an exposition where some pixels are saturated. a-b-c-d-e-f. The bands of the molecular weight marker were visualized by white light and copied on the blots by superposition.


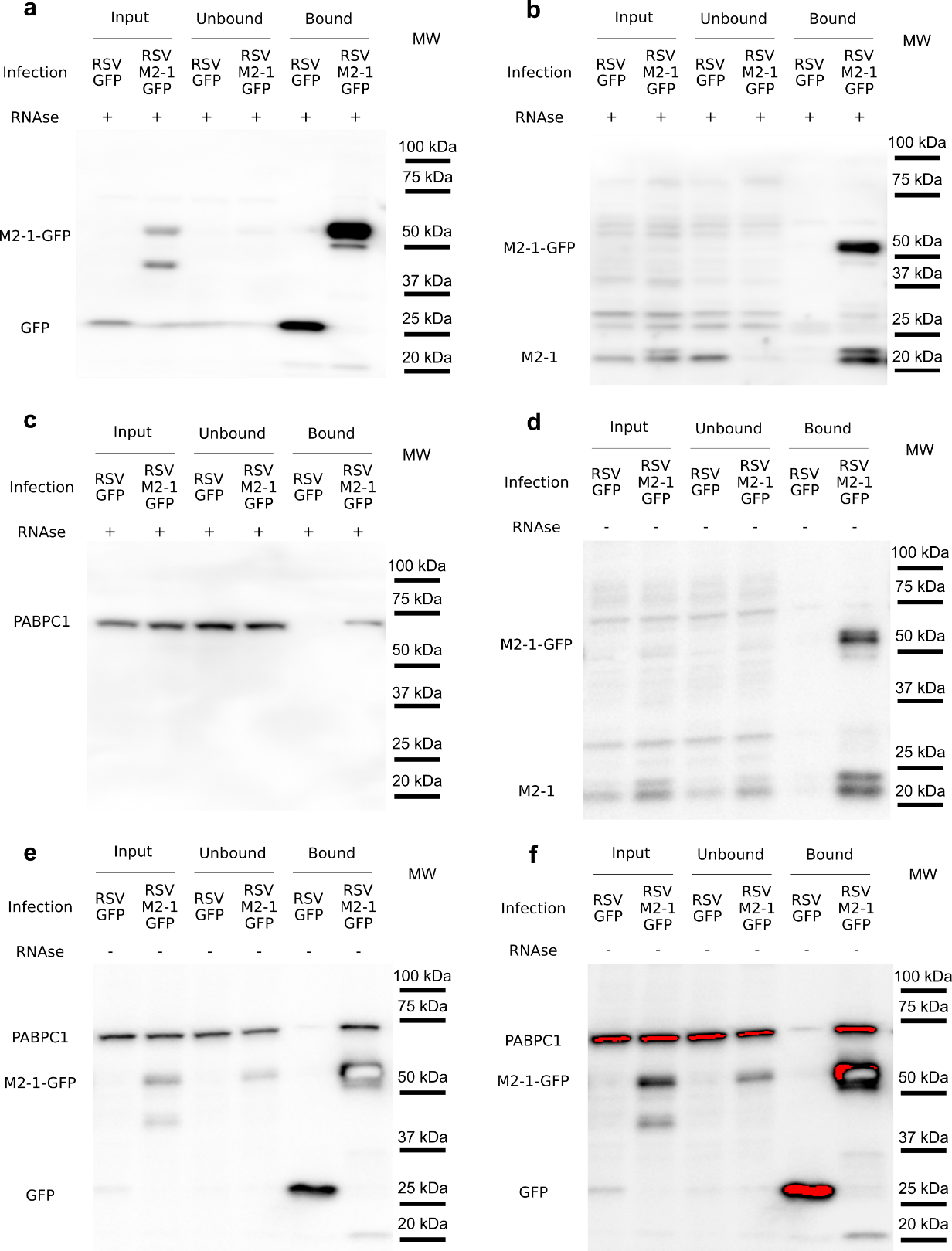


Supplementary Figure S5: Interaction of PABPC1 with M2-1 expressed in an infectious context (complement to Figure 5a)

HEp-2 cells were infected with either RSV-M2-1-GFP or RSV-GFP (control) for 14h and then subjected to co-IP with GFP antibody. RNAse A was added (a,b,c) or not (d,e,f) during the lysis step. The whole cell lysate (input) and the fraction bound to the beads (bound) were subjected to SDS-PAGE. a. The membrane was probed by an antibody directed against GFP. b. The membrane was probed by an antibody directed against M2-1. c. The membrane was probed by an antibody directed against PABPC1. d. The membrane was probed by an antibody directed against M2-1. e-f. The membrane was probed by antibodies directed against PABPC1 and GFP. a-b-c-d-e. Antibody signals were visualized by luminescence, at an exposition where no pixel was saturated. Hollow bands result from an excess of substrate and not overexposition. f. Antibody signals were visualized by luminescence, at an exposition where some pixels are saturated (red). a-b-c-d-e-f. The bands of the molecular weight marker were visualized by white light and copied on the blots by superposition.


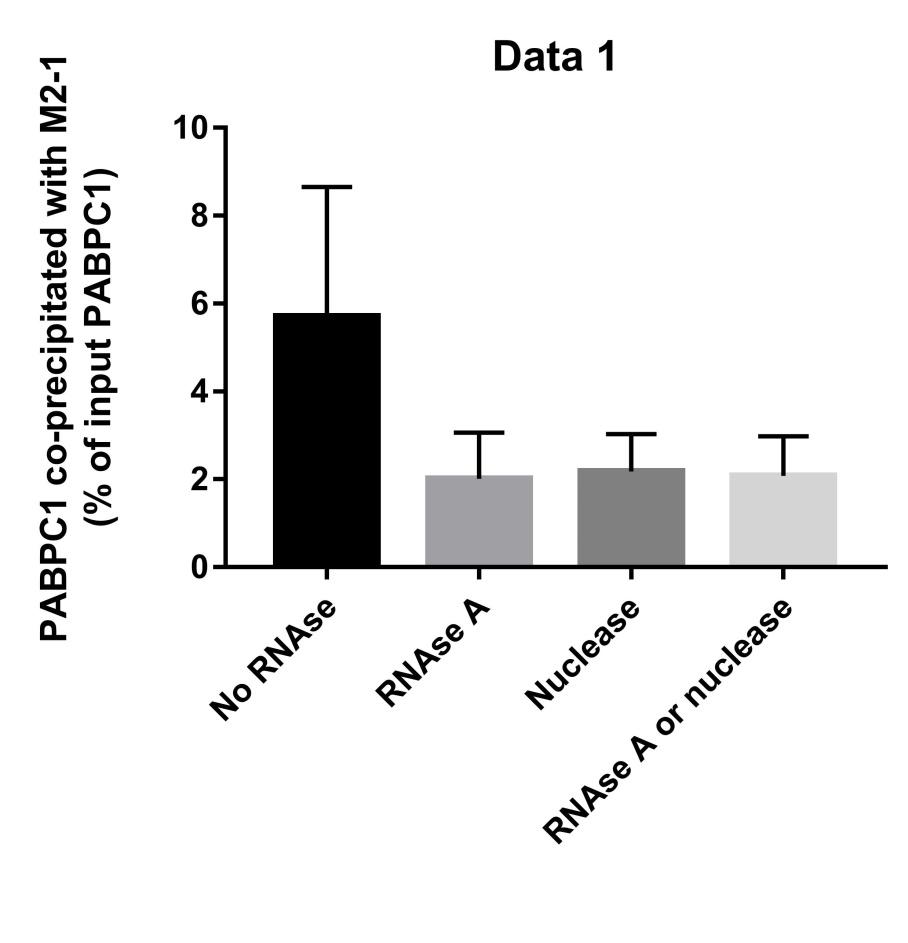


Supplementary Figure S6: Amount of PABPC1 co-immunoprecipitated with M2-1 in an infectious context

HEp-2 cells were infected with RSV-M2-1-GFP for 14h and then subjected to co-IP with GFP antibody. RNAse A or broad spectrum nuclease were added or not during the lysis step. The whole cell lysate (input) and the fraction bound to the beads (bound) were subjected to SDS-PAGE. The membrane was probed by an antibody directed against PABPC1, and the antibody’s signal was visualized by luminescence, at an exposition where no pixel was saturated. Relative quantification of band intensities in the bound fraction was performed using the Image Lab software (Bio-Rad), using the Input fraction as reference. The data shown (mean $\boldsymbol{\pm}$ s.d.) represents 6 (No RNAse), 4 (RNAse A), 3 (nuclease) or 7 (RNAse A or nuclease) independent experiments.


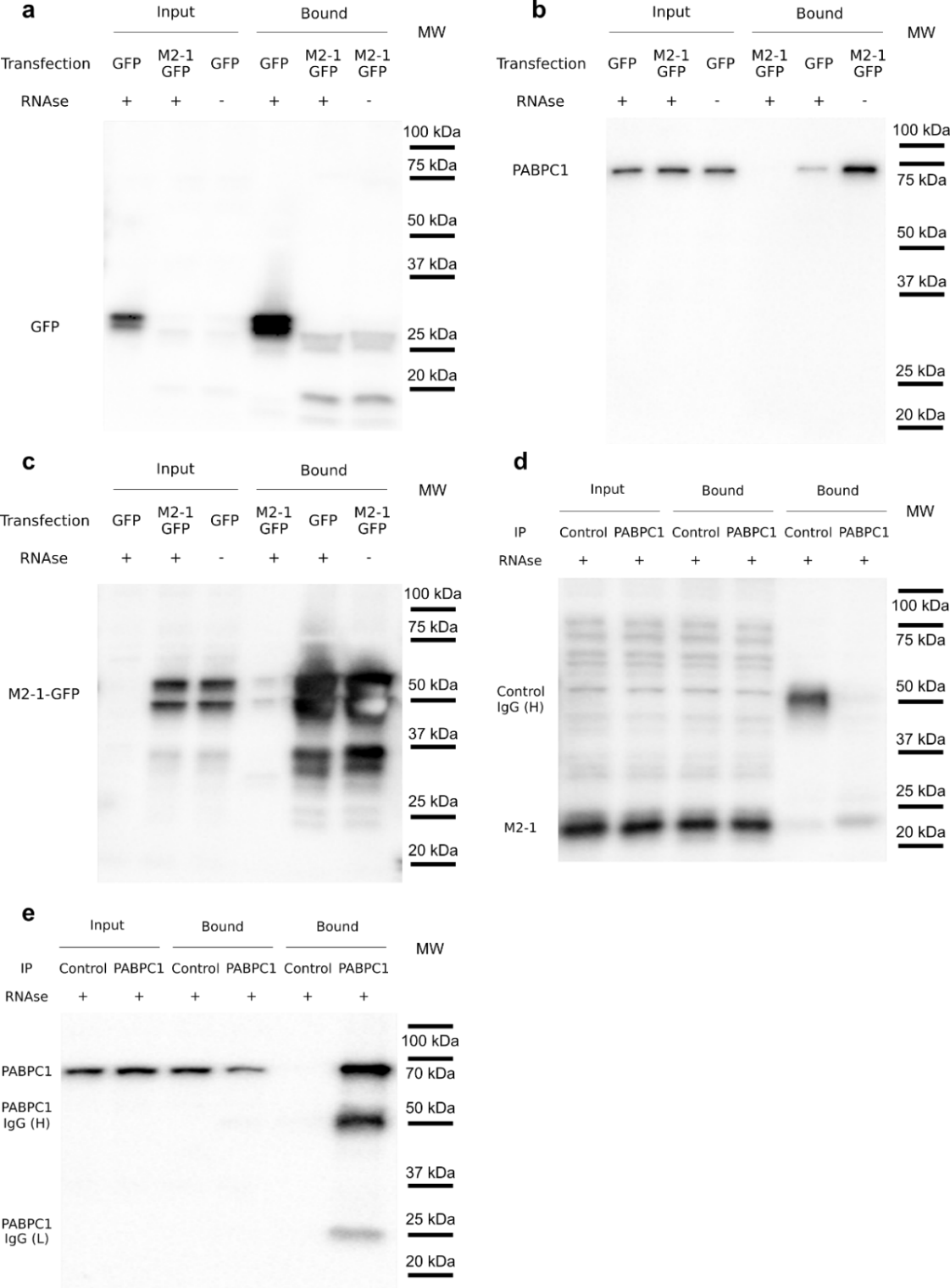


Supplementary Figure S7: Interaction of PABPC1 with M2-1 expressed either alone or in an infectious context (complement to Figure 5b and 5c)

a-b-c. HEp-2 cells were transfected with either M2-1-GFP or GFP (control) for 24h, and then subjected to co-IP with an anti-GFP antibody. The whole cell lysate (input) and the fraction bound to the beads (bound) were subjected to SDS-PAGE. a. The membrane was probed by an antibody directed against GFP. b. The membrane was probed by an antibody directed against PABPC1. c. The membrane was probed by an antibody directed against M2-1. d-e. HEp-2 cells were infected with wild type RSV for 14h, and then subjected to co-IP with an antibody directed against either PABPC1 or a non-relevant protein (IMPDH2). RNAse A was added during the lysis step. The whole cell lysate (input) and the fraction bound to the beads (bound) were subjected to SDS-PAGE. d. The membrane was probed by an antibody directed against M2-1. e. The membrane was probed by an antibody directed against PABPC1. a-b-c-d-e. Antibody signals were visualized by luminescence, at an exposition where no pixel was saturated. Hollow bands result from an excess of substrate and not overexposition. The bands of the molecular weight marker were visualized by white light and copied on the blots by superposition


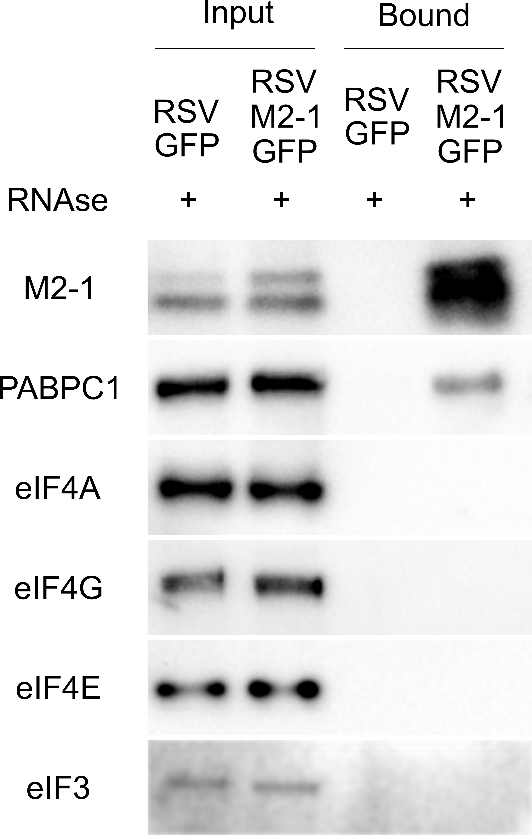


Supplementary Figure S8: Absence of interaction between M2-1 and other proteins of the translation initiation complex.

HEp-2 cells were infected with either RSV M2-1-GFP or RSV-GFP (control) for 14h, then subjected to co-immunoprecipitation with GFP antibody. RNAse A was added during the lysis step. Western blot was then performed with antibodies directed against M2-1, PABPC1, eIF4A, eIF4E, eIF3 and eIF4G.

**Supplementary Movie S1: PABPC1 and M2-1 dynamic**

Time-lapse microscopy of M2-1-GFP and PABPC1-Cherry in HEp-2 cells simultaneously infected by RSV-M2-1-GFP and transfected with PABPC1-Cherry. At 24h p.i., cells were imaged every minute in a chamber heated at 37°C, with a Olympus FV3000 confocal microscope. The resulting movies were visualized under the Icy software. A representative movie out of 4 independent experiments is shown (5 Frames per second).


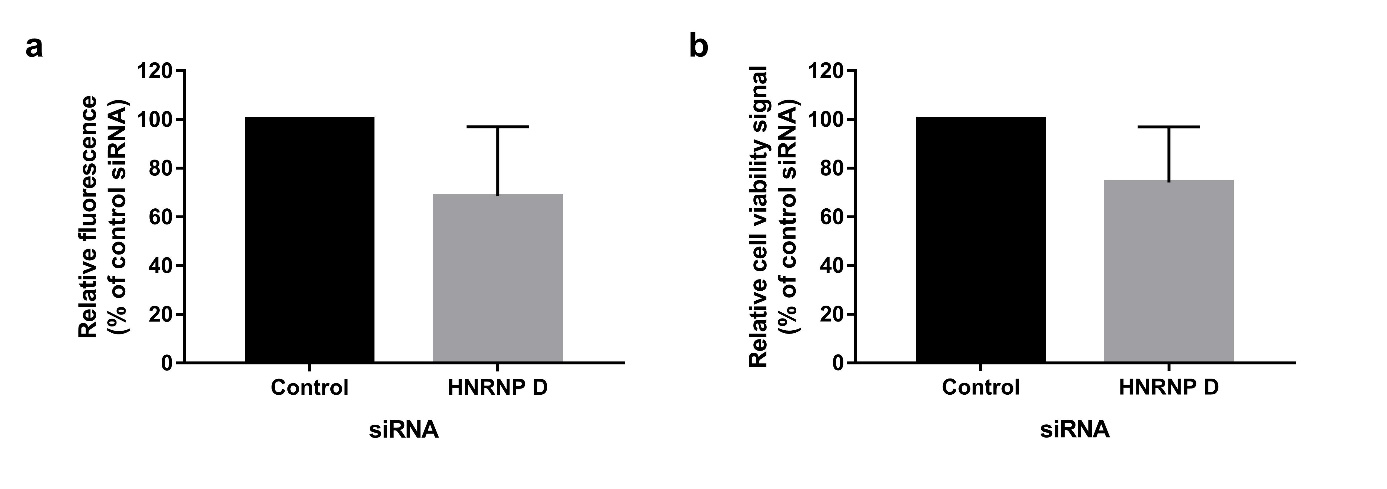


Supplementary Figure S9: hnRNP D silencing by siRNA.

A549 cells were treated by siRNA targeting hnRNP D and non-targeting siRNA (control) and infected after 24h by RSV Cherry at low MOI. A non-targeting siRNA was used as control. a. Cherry fluorescence was measured at 48h p.i and expressed as a percentage of the control siRNA's signal. The data shown (mean $\boldsymbol{\pm}$ s.d.) represents 4 independent experiments, with each point performed in triplicate. No significant difference between the two conditions was found by two-tailed paired t-test (alpha=0.05) using GraphPad Prism software (p=0.0710). The normality of the data was tested with a Shapiro-Wilk normality test (alpha=0.05) using GraphPad Prism software (p=0.4202). b. Cell viability signal was measured at 0 and 48h p.i using Cell Titer Glo kit (Promega). The cell viability signal was obtained as a luminescence measure, and expressed as a percentage of the control siRNA's signal. The data shown (mean $\boldsymbol{\pm}$ s.d.) represents 4 independent experiments, with each point performed in triplicate. No significant difference between the two conditions was found by two-tailed paired t-test (alpha=0.05) using GraphPad Prism software (p=0.0942). The normality of the data was tested with a Shapiro-Wilk normality test (alpha=0.05) using GraphPad Prism software (p=0.7908).

Supplementary Table S5: Primers used in qPCR

| **Target** | **Primer** |  | **Sequence** | **Efficiency^a^** |
| --- | --- | --- | --- | --- |
| **GAPDH** | GAPD-For |  | GGAGTCAACGGATTTGGTCG | 1.91 |
|  | GAPD-Rev |  | CGGTGCCATGGAATTTGCCA |  |
| **PABPC1** | PABPC1-For |  | GAGGCGATGCTCTACGAGAA | 1.96 |
|  | PABPC1-Rev |  | GCTGGAAGTTCACATACGCG |  |
| **RSV N** | N-For |  | GGTGAAGCAGGATTCTACCATA | 1.92 |
|  | N-Rev |  | GTGTACCTCTGTACTCTCCC |  |
| **RSV L** | L-For |  | TATCGTGAGTTTCGGTTGCCTA | 1.88 |
|  | L-Rev |  | TCTTCTTGATTTATCACTCTCGGA |  |
| **UBC^b^** | UBC-For |  | TGTGGATCGCTGTGATCGTC | 1.94 |
|  | UBC-Rev |  | GGAGGGATGCCTTCCTTATC |  |
| **PPIA^b^** | PPIA-For |  | CTGCACTGCCAAGACTGAGT | 1.88 |
|  | PPIA-Rev |  | GTCCACAGTCAGCAATGGTG |  |
| **ACT B^b^** | ACT B-For |  | CATGGGTCAGAAGGATTCCTA | 1.94 |
|  | ACTB-Rev |  | TAGAAGGTGTGGTGCCAGATT |  |

^a^The efficiency of each primer pair was calculated as described in (Pfaffl 2001), using 4-fold serial dilutions of cDNA obtained by reverse transcription of whole cell lysate.

^b^Used as a reference gene.
